# *In vitro* and *in vivo* Human Metabolism of (*S*)-[^18^F]Fluspidine – A Radioligand for Imaging σ_1_ Receptors With Positron Emission Tomography (PET)

**DOI:** 10.3389/fphar.2019.00534

**Published:** 2019-06-13

**Authors:** Friedrich-Alexander Ludwig, Steffen Fischer, Richard Houska, Alexander Hoepping, Winnie Deuther-Conrad, Dirk Schepmann, Marianne Patt, Philipp M. Meyer, Swen Hesse, Georg-Alexander Becker, Franziska Ruth Zientek, Jörg Steinbach, Bernhard Wünsch, Osama Sabri, Peter Brust

**Affiliations:** ^1^Department of Neuroradiopharmaceuticals, Helmholtz-Zentrum Dresden-Rossendorf, Institute of Radiopharmaceutical Cancer Research, Leipzig, Germany; ^2^ABX Advanced Biochemical Compounds GmbH, Radeberg, Germany; ^3^Department of Pharmaceutical and Medicinal Chemistry, University of Münster, Münster, Germany; ^4^Department of Nuclear Medicine, Leipzig University, Leipzig, Germany; ^5^Integrated Research and Treatment Center (IFB) Adiposity Diseases, Leipzig University, Leipzig, Germany

**Keywords:** sigma-1 receptors, fluspidine, positron emission tomography, radiometabolites, liquid chromatography-mass spectrometry, liver microsomes

## Abstract

(*S*)-[^18^F]fluspidine ((*S*)-[^18^F]**1**) has recently been explored for positron emission tomography (PET) imaging of sigma-1 receptors in humans. In the current report, we have used plasma samples of healthy volunteers to investigate the radiometabolites of (*S*)-[^18^F]**1** and elucidate their structures with LC-MS/MS. For the latter purpose additional *in vitro* studies were conducted by incubation of (*S*)-[^18^F]**1** and (*S*)-**1** with human liver microsomes (HLM). *In vitro* metabolites were characterized by interpretation of MS/MS fragmentation patterns from collision-induced dissociation or by use of reference compounds. Thereby, structures of corresponding radio-HPLC-detected radiometabolites, both *in vitro* and *in vivo* (human), could be identified. By incubation with HLM, mainly debenzylation and hydroxylation occurred, beside further mono- and di-oxygenations. The product hydroxylated at the fluoroethyl side chain was glucuronidated. Plasma samples (10, 20, 30 min p.i., *n* = 5-6), obtained from human subjects receiving 250–300 MBq (*S*)-[^18^F]**1** showed 97.2, 95.4, and 91.0% of unchanged radioligand, respectively. In urine samples (90 min p.i.) the fraction of unchanged radioligand was only 2.6% and three major radiometabolites were detected. The one with the highest percentage, also found in plasma, matched the glucuronide formed *in vitro*. Only a small amount of debenzylated metabolite was detected. In conclusion, our metabolic study, in particular the high fractions of unchanged radioligand in plasma, confirms the suitability of (*S*)-[^18^F]**1** as PET radioligand for sigma-1 receptor imaging.

## Introduction

**FIGURE 1:**
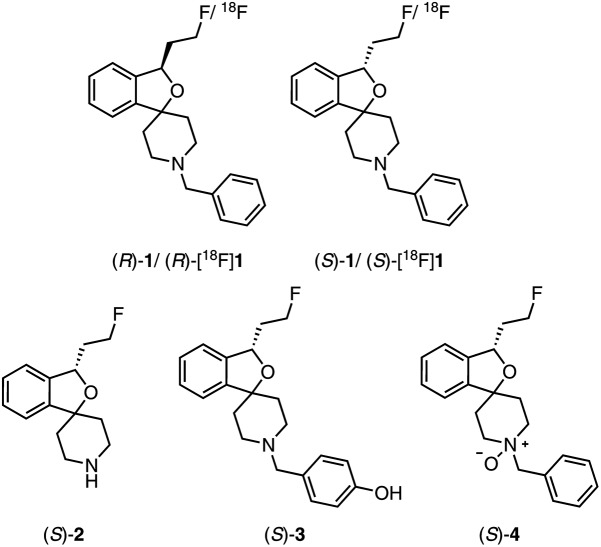
Chemical structures of both fluspidine and [^18^F]fluspidine enantiomers ((*S*)- and (*R*)-**1** and (*S*)- and (*R*)-[^18^F]**1**) and selected *in vitro* metabolites detected after incubation of (*S*)-**1** with rat liver microsomes (RLM). Synthesized racemic compounds *rac*-**2**, *rac*-**3**, and *rac*-**4** were used as reference compounds in this study (Holl et al., 2013).

The two enantiomers (*S*)- and (*R*)-[^18^F]fluspidine ((*S*)- and (*R*)-[^18^F]1′ -benzyl-3-(2-fluoroethyl)-3*H*-spiro[2-benzofuran-1,4′ -piperidine], (*S*)-**1** and (*R*)-**1**, Figure 1) are radioligands which have been developed for positron emission tomography (PET) imaging of sigma-1 receptors (S1R) in human (Weber et al., 2017).

S1R are expressed in the central nervous system (CNS) as well as in peripheral tissues (Rousseaux and Greene, 2015). They are located in both the plasma membrane and the mitochondria-associated membrane of the endoplasmatic reticulum, where they are involved in different physiological and pathophysiological processes, e.g., regulations of ion channels and neurotransmitter receptors. The role of S1R has been increasingly recognized as they serve as a novel promising target for the therapy of, e.g., cancer (Tesei et al., 2018) or cardiovascular diseases (Stracina and Novakova, 2018) as well as of CNS disorders (Jia et al., 2018; Penke et al., 2018), notably Alzheimer’s disease (Maurice and Goguadze, 2017), Parkinson’s disease and depression (Fishback et al., 2010). PET offers the possibility for earlier diagnoses or monitoring of therapeutic processes (Brust et al., 2014b; Honer et al., 2014; Barthel et al., 2015; Song et al., 2018; Jia et al., 2019).

(*S*)-**1** and (*R*)-**1** are derived from a structural optimization process for the purpose of PET of spirocyclic piperidines which possess high affinity toward S1R and selectivity over a variety of other receptors (Maier and Wünsch, 2002a,b; Große Maestrup et al., 2009a, 2011; Maisonial et al., 2011; Holl et al., 2014; Nakane et al., 2018). Attempts to improve the synthesis of (*S*)-**1** or (*R*)-**1** (Holl et al., 2013) culminated in a recently published synthesis route, which circumvents chiral HPLC separation by an enantioselective reduction step and additionally forms the basis for further easily accessible structural modifications (Nakane et al., 2018).

Labeling with fluorine-18 has been described for racemic [^18^F]fluspidine (rac-[^18^F]**1**) and its [^18^F]fluoroalkyl homologs (Große Maestrup et al., 2009b; Fischer et al., 2011; Maisonial et al., 2011, 2012; Maisonial-Besset et al., 2014) as well as for enantiomerically pure (*S*)- and (*R*)-[^18^F]**1**, both made available by automated synthesis procedures suitable for application in human studies (Brust et al., 2014a; Kranz et al., 2016).

As a prerequisite for clinical studies both enantiomeric radioligands have been investigated in animals (mice, pigs, and monkeys) with regard to radiation dosimetry and toxicity (Kranz et al., 2016) and regarding specificity and kinetic modeling (Brust et al., 2014a; Baum et al., 2017). The studies revealed that both (*S*)-[^18^F]**1** and its *R-*enantiomer appeared to be suitable for S1R imaging in humans. First clinical trials using (*S*)-[^18^F]**1** to quantify the S1R availability in patients with major depressive disorder and Huntingtons’s disease have been performed (EudraCT Numbers: 2014-005427-27, 2016-001757-41).

The present study reports on accompanying investigations on the metabolic fate of (*S*)-[^18^F]**1**. Beside determination of the fraction of unchanged radioligand in plasma for providing an arterial input function, which enables reliable analysis of PET data, a characterization of the formed radiometabolites (metabolites bearing a radioactive nuclide) is of interest (Pawelke, 2005). However, the low mass of radioligands in plasma samples, which is related to the use of substances with high molar activities in PET studies, usually prevents a direct structural elucidation. This difficulty can be overcome by means of *in vitro* investigations (Jia and Liu, 2007; Asha and Vidyavathi, 2010) supported by liquid chromatography-mass spectrometry (LC-MS) or -tandem mass spectrometry (LC-MS/MS) (Bier et al., 2006; Amini et al., 2013; Ludwig et al., 2016, 2018) to enable interpretation of radiometabolite profiles obtained by radio-HPLC, e.g., during a clinical study.

In a detailed study the metabolism of racemic fluspidine (rac-**1**) as well as its fluoroalkyl homologs and the corresponding ^18^F-radioligands have been investigated covering incubations with rat liver microsomes (RLM) and *in vivo* studies in mice, respectively (Wiese et al., 2016). Similarly, enantiomerically pure (*S*)-**1** as well as (*R*)-**1** have been investigated *in vitro* and structural elucidation with LC-MS has been conducted for metabolites formed by incubations with RLM under conditions for oxygenation (Holl et al., 2013). Mainly formed metabolites of (*S*)-**1** resulted from debenzylation ((*S*)-**2**), hydroxylation at the phenyl ring ((*S*)-**3**), and *N*-oxidation ((*S*)-**4**) and their structures are provided in Figure 1. Further, hydroxylation at the piperidine moiety and at the fluoroethyl side chain was observed, as well as the formation of three di-oxygenated degradation products. For the corresponding radioligand (*S*)-[^18^F]**1** metabolic stability in piglets has been reported, whereby in plasma 48% of the radioligand remained unchanged at 16 min post injection (Brust et al., 2014a). However, structures of radiometabolites formed *in vivo* have not been elucidated so far.

In the present study, the metabolism of (*S*)-[^18^F]**1** was investigated using human liver microsomes (HLM) to structurally characterize *in vitro* metabolites. The *in vitro* metabolite profile was used to identify radiometabolites in human plasma und urine after administration of (*S*)-[^18^F]**1** thereby providing knowledge about the metabolic pathways of this radioligand in the human body.

## Materials and Methods

### Chemicals and Reagents

Acetonitrile (gradient grade) was purchased from VWR International (Darmstadt, Germany). Acetonitrile and water (both for LC-MS) were purchased from Fisher Scientific (Schwerte, Germany). Ammonium acetate (for HPLC) was purchased from Acros Organics (Geel, Belgium). Ammonium acetate (LC-MS), testosterone, 4-nitrophenol, β-nicotinamide adenine dinucleotide 2′-phosphate reduced tetrasodium salt (NADPH), uridine 5′-diphosphoglucuronic acid trisodium salt (UDPGA), alamethicin, MgCl_2_, and β-glucuronidase were purchased from Sigma-Aldrich (Merck KGaA, Darmstadt, Germany). GIBCO human liver microsomes (HLM, pooled donors, 20 mg/mL) were purchased from Life Technologies (Thermo Fisher Scientific, Darmstadt, Germany). Dulbecco’s phosphate-buffered saline (PBS) (without Ca^2+^, Mg^2+^) was purchased from Biochrom (Berlin, Germany). (*S*)-**1**, *rac*-**2**, *rac*-**3**, and *rac*-**4** were synthesized as reported in literature (Holl et al., 2013).

### Radiosynthesis of (*S*)-[^18^F]1

(*S*)-[^18^F]**1** was synthesized by nucleophilic no-carrier-added (n.c.a.) ^18^F-substitution of the tosyl precursor under GMP conditions for human application according to a procedure previously reported (Fischer et al., 2011; Maisonial-Besset et al., 2014; Kranz et al., 2016).

### Incubations With Human Liver Microsomes (HLM)

#### Time- and Concentration-Dependent Metabolic Transformation of (*S*)-[^18^F]1

Incubations had a final volume of 250 μL and were performed in PBS (pH 7.4) as follows (Ludwig et al., 2018), with final concentrations as provided in brackets. Respective amounts of a solution of (*S*)-**1** of 0.1 mg/mL [<1 μM (referring to estimated molar activity; no addition of carrier), 2, and 20 μM] in acetonitrile were put into test tubes and the solvent was evaporated using the DB-3D TECHNE Sample Concentrator (Biostep, Jahnsdorf, Germany) at room temperature under a stream of nitrogen. PBS and HLM (1 mg/mL) and ∼5 MBq (*S*)-[^18^F]**1** (molar activity: 17 GBq/μmol, at the start of incubations) in 100 μl PBS were added, mixed vigorously, and pre-incubated at 37°C for 3 min. Analogously pre-incubated NADPH (2 mM) was added and mixtures were shaken gently at 37°C using the BioShake iQ (QUANTIFOIL Instruments, Jena, Germany). After 0, 2, 5, 10, 15, 20, 30, 40, 50, and 60 min, samples of 20 μL were taken and added to 80 μL cold acetonitrile (-20°C), followed by vigorous shaking (30 s), cooling on ice (4 min), and centrifugation at 14,000 rpm (10 min). Supernatants (90 μL) were diluted with water (30 μL) and immediately analyzed by radio-HPLC (system II).

#### Incubation of (*S*)-[^18^F]1 and Non-radioactive References for Identification of *in vitro* Metabolites and Radiometabolites

Incubations with (*S*)-[^18^F]**1**, (*S*)-**1**, and *rac***-2**, *rac***-3**, and *rac***-4** as substrate, had a final volume of 250 μL and were performed in PBS (pH 7.4) in duplicate. In the following, the final concentrations are provided in brackets. For incubations of (*S*)-[^18^F]**1** together with (*S*)-**1** under conditions for oxidation and glucuronidation, a solution of (*S*)-**1** of 0.1 mg/mL (2 μM) in acetonitrile was put into test tubes and the solvent was evaporated using the DB-3D TECHNE Sample Concentrator (Biostep) at room temperature under a stream of nitrogen. HLM (1 mg/mL) and alamethicin (50 μg/mL, from methanolic solution) were mixed (Fisher et al., 2000), kept on ice for 15 min and added to the test tubes. PBS, ∼5 MBq (*S*)-[^18^F]**1** in 20 μL PBS, and MgCl_2_ (2 mM) were added, mixed vigorously and the mixture was pre-incubated at 37°C for 3 min. After addition of analogously pre-incubated NADPH (2 mM) and UDPGA (5 mM), the incubations were continued by gentle shaking at 37°C for 120 min using the BioShake iQ (QUANTIFOIL Instruments). After termination by adding 1.0 mL of cold acetonitrile (-20°C) and vigorous mixing for 30 s, the mixtures were stored at 4°C for 5 min. After centrifugation at 14,000 rpm (Eppendorf Centrifuge 5424) for 10 min and the concentration of the supernatants at 50°C under a flow of nitrogen (DB-3D TECHNE Sample Concentrator) residual volumes of 40–70 μL each were obtained, which were reconditioned by adding water to provide samples of 100 μL, which were immediately analyzed by radio-HPLC and stored at 4°C until analysis by LC-MS/MS. HLM incubations of *rac*-**2**, *rac*-**3**, and *rac*-**4**, as substrate were performed in similar manner. Incubations without HLM, NADPH, UDPGA, and substrates, respectively, were performed as negative controls and to provide conditions only for oxygenation and not glucuronidation, and vice versa. As positive controls, testosterone (for oxygenation) and 4-nitrophenol (for glucuronidation) were incubated at appropriate concentrations, similarly to the protocol described above. Complete conversions of both were confirmed by RP-HPLC analyses with UV detection.

In order to investigate the cleavage of formed glucuronides (see section “*β*-Glucuronidase Cleavage of Microsomal-Formed Metabolite **M12**”), microsomal incubation of (*S*)-**1** was performed in quadruplicate with higher amounts of the substrate and the reagents as follows (final concentrations in brackets): (*S*)-**1** (200 μM), HLM (2 mg/mL), NADPH (4 mM), MgCl_2_ (5 mM), alamethicin (0.1 mg/mL), UDPGA (10 mM). After addition of acetonitrile, resulting mixtures were combined and further processing was proceeded as described above. The obtained sample was separated chromatographically using the HPLC system I (Section “Radio-HPLC”) with UV monitoring at 210 nm. The constituent eluting at 24.6 min (**M12**) was collected manually and further used for cleavage experiments as described in Section “*β*-Glucuronidase Cleavage of Microsomal-Formed Metabolite **M12**.”

### *β*-Glucuronidase Cleavage of Microsomal-Formed Metabolite M12

To 10 μL of a solution of metabolite **M12** (Section “Incubation of (*S*)-[^18^F]1 and Non-radioactive References for Identification of *in vitro* Metabolites and Radiometabolites”), 32 μL acetate buffer (NaOAc/AcOH, 100 mM, pH 4.5–5.0), and 8 μL *β*-glucuronidase from *Helix pomatia* Type H-3 (aqueous solution, ≥90,000 units/mL, final: ≥14,400 units/mL) were added. The mixture was shaken gently at 37°C for 2 h, then 50 μL of cold acetonitrile (4°C) were added and mixed vigorously. After centrifugation at 14,000 rpm (Eppendorf Centrifuge 5424) for 15 min, 40 μL of the supernatant was diluted with 60 μL of water and analyzed by LC-MS/MS monitoring the MRM transitions *m/z* 518.2/342.2 (as for glucuronides of mono-oxygenated metabolites, e.g., **M12**) and 342.2/91.1 (as for mono-oxygenated metabolites, e.g., **M5**).

### Investigation of the Metabolism of (*S*)-[^18^F]1 in Humans

All investigations were conducted in the framework of an approved and registered clinical study (EudraCT Number: 2014-005427-27).

After injection of 244.6-290.4 MBq (mean: 265.5 MBq) of (*S*)-[^18^F]**1** into 8 healthy controls arterial blood samples (∼16 mL) were withdrawn at 10, 20, and 30 min. The samples were collected directly into S-Monovettes^®^ 9 mL K3E (SARSTEDT, Nümbrecht, Germany) and stored on ice. After 90 or 120 min, urine (∼8 mL) was collected and stored on ice. Plasma was obtained by centrifugation of blood samples at 7,000 rpm (UNIVERSAL 320 R, Hettich, Germany) for 7 min. Protein precipitation and extraction with acetonitrile was conducted as follows. Method A: 10 × 1.6 mL cold acetonitrile (-35°C) were added to 10 × 400 μL plasma, shaken for 3 min, cooled at 4°C for 5 min and centrifuged at 7,000 rpm (Eppendorf Centrifuge 5424) for 5 min. Supernatants were collected and the precipitates were extracted with 1.6 mL acetonitrile each. The combined supernatants were concentrated at 70°C under a flow of nitrogen (Sample Concentrator DB-3D TECHNE) to provide residual volumes of 40–70 μL, which were reconditioned by adding water to obtain samples of 100 μL, which were immediately analyzed by radio-HPLC (system I). Method B: similar to method A, using 2 × 8 mL cold acetonitrile (-35°C) and 2 × 2 mL plasma. After centrifugation, the precipitates were extracted with 2 × 4 mL acetonitrile.

For monitoring of the efficiency of extraction for (*S*)-[^18^F]**1** and its radiometabolites, the precipitants and aliquots of plasma and supernatants were taken and measured in a calibrated gamma counter (Wallac WIZARD 3, Perkin Elmer, Shelton, CT, United States). The recovery in % was calculated as follows: recovery = activity_supernatant_/(activity_supernatant_ + activity_precipitate_) × 100%. Urine samples were analyzed without any pre-treatment.

### Radio-HPLC

System I: Analyses were performed on a JASCO LC-2000 system (JASCO Labor- und Datentechnik, Gross-Umstadt, Germany) including a UV-2070 UV–VIS detector (monitoring at 210 nm) online with a GABI Star radioactivity flow detector (raytest Isotopenmessgeräte, Straubenhardt, Germany) with a NaI detector (2 × 2″ pinhole, 16 mm × 30 mm). Chromatographic separations were achieved using a Multospher 120 RP 18 AQ-5μ-column, 250 mm × 4.6 mm, 5 μm, including pre-column, 10 mm × 4 mm (CS-Chromatographie Service, Langerwehe, Germany). The solvent system consisted of eluent A: water/acetonitrile, 95:5 (v/v), containing NH_4_OAc (20 mM) and eluent B: water/acetonitrile, 20:80 (v/v), containing NH_4_OAc (20 mM). Linear gradient elution (% acetonitrile) at a flow rate of 1.0 mL/min: 0–5 min, 5%; 5–55 min, 5–80%; 55–65 min, 80%; 65–66 min, 80–5%; 66–76 min, 5%.

System II: Analyses of samples from microsomal incubations to determine time and concentration dependency of microsomal transformation were performed on a JASCO X-LC system (JASCO Labor- und Datentechnik) equipped with a UV/Vis detector UV-2070 (monitoring at 210 nm) and a radioactivity flow detector GABI Star (raytest Isotopenmessgeräte, Straubenhardt, Germany) including NaI detector (2 × 2″ pinhole, 16 mm × 30 mm). Chromatographic separations were achieved using a Multospher 120 RP 18 AQ-3μ-column, 125 mm × 3 mm, 3 μm, including pre-column, 10 mm × 3 mm (CS-Chromatographie Service) at a column temperature of 20°C, at a flow rate of 0.7 mL/min and a runtime of 5.0 min. The isocratic solvent system consisted of water/acetonitrile 40:60 (v/v), containing NH_4_OAc (20 mM). Unchanged (*S*)-[^18^F]**1** eluted at a retention time of 4.1 min, whilst formed radiometabolites eluted at 0.6–2.2 min. The percentages of unchanged (*S*)-[^18^F]**1** were calculated from HPLC data as follows: Fraction of (S) - [^18^F]**1**(%) = Peak area_(S)-[18F]**1**_/(Peak area_(S)-[18F]**1**_ + Peak area_radiometabolites_) × 100%

### LC-MS/MS Analyses

Analyses were performed on an Agilent 1260 Infinity Quaternary LC system (Agilent Technologies, Böblingen, Germany) coupled with a QTRAP 5500 hybrid linear ion-trap triple quadrupole mass spectrometer (AB SCIEX, Concord, ON, Canada). Data were acquired and processed using Analyst software (Version 1.6.1, AB SCIEX) and for further data processing OriginPro 2017G (OriginLab, Northampton, MA, United States) was used. For chromatographic separations a Multospher 120 RP 18 AQ-3μ-column, 125 mm × 3 mm, 3 μm, including pre-column, 10 mm × 3 mm (CS-Chromatographie Service) was used. The solvent system consisted of eluent A: water containing NH_4_OAc (2 mM) and eluent B: water/acetonitrile, 20:80 (v/v), containing NH_4_OAc (2 mM). Linear gradient elution (% acetonitrile) at a flow rate of 0.7 mL/min, at 40°C: 0–10 min, 5–80%; 10–12 min, 80%; 12–16 min, 5%. The mass spectrometer was operated in positive electrospray ionization mode with the following parameters: curtain gas (CUR) 35, collision gas (CAD) medium, ion spray voltage (IS) 5500, temperature (TEM) 550, ion source gas 1 (GS1) 60, and ion source gas 2 (GS2) 50.

For multiple reaction monitoring (MRM) scan type: appropriate MRM transitions, scan time 40 ms; declustering potential (DP) 110; entrance potential (EP) 10; collision energy (CE) 40; collision cell exit potential (CXP) 10.

For enhanced product ion (EPI) scan type: products of selected *m/z* values, scan rate 10000 Da/s, dynamic fill time, CAD high, and further parameters as used for MRM scans. In EPI chromatograms obtained, a range of background was selected manually and subtracted from ranges of interest to result in EPI spectra as provided in the Supplementary Material.

For the MS^3^ scan type the excitation energy (AF2) was optimized prior to data acquisition.

## Results and Discussion

### Investigation of the Metabolism of (*S*)-[^18^F]1 and (*S*)-1 *in vitro* Using Human Liver Microsomes (HLM)

#### Time- and Concentration-Dependent Microsomal Transformation

In order to obtain basic information about the metabolic stability *in vitro*, the time course of the degradation of (*S*)-[^18^F]**1** was investigated in presence of different concentrations of (*S*)-**1** [no-carrier-added (n.c.a., <1 μM) and carrier-added: 2 and 20 μM]. For that purpose, incubations were performed in PBS with HLM and NADPH at 37°C. At defined time points (until 60 min) samples were taken and added to cold acetonitrile to terminate the incubations. After centrifugation, the fractions of remaining (*S*)-[^18^F]**1** were determined by radio-HPLC.

**FIGURE 2:**
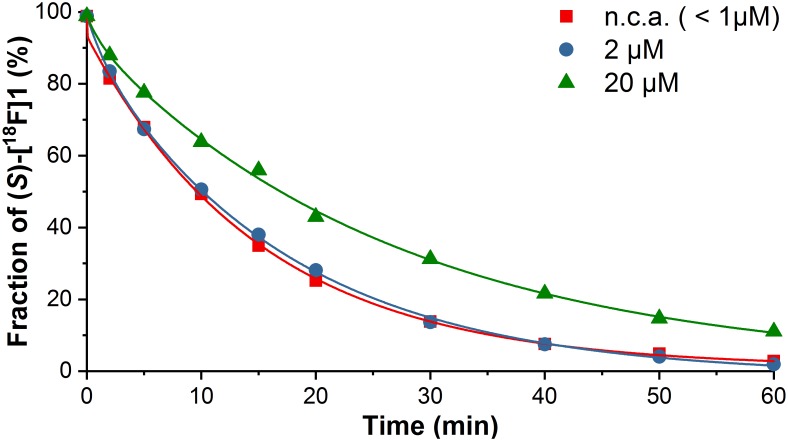
Microsomal metabolic transformation of (*S*)-[^18^F]**1** at different concentrations of carrier ((*S*)-**1**) by HLM and NADPH in PBS at 37°C (Section “Time- and Concentration-Dependent Metabolic Transformation of (*S*)-[^18^F]**1**”) determined by radio-HPLC (system II in Section “Radio-HPLC”).

The time course of degradation is illustrated in Figure 2. The fraction of (*S*)-[^18^F]**1** represents the fraction of both remaining (*S*)-[^18^F]**1** and (*S*)-**1**, with regard to formed (radio)metabolites. After 60 min, only 3% of unchanged (*S*)-[^18^F]**1** (n.c.a.) was detectable, whilst after 30 min incubation 14% of unchanged radioligand was present. Similar percentages were found using 2 μM (*S*)-**1**, whereas at a concentration of 20 μM the metabolic degradation was diminished, which can be explained by saturation of the degrading cytochrome P450 enzyme system. 50% of unchanged (*S*)-[^18^F]**1** could be detected (a) after 10 min for both n.c.a. and a concentration of 2 μM and (b) after 17 min for a concentration of 20 μM of added (*S*)-**1**. Therefore, for most carrier-added experiments a concentration of 2 μM was chosen.

The metabolic stability of *rac*-**1**, (*S*)-**1**, and (*R*)-**1** have previously been studied *in vitro* using RLM in presence of NADPH. After 30 min, *rac*-**1** showed the lowest stability (∼13%) among the fluoroalkyl homologs tested (Wiese et al., 2016), whereas 73% of intact (*S*)-**1** was still present after the same incubation time (Holl et al., 2013). Compared with our results from HLM (14%) this might suggest that (*S*)-**1** has higher stability in rats than in human. However, for RLM incubations, beside different incubation conditions, (*S*)-**1** was used at a concentration of 320 μM, which is a substantially higher concentration than the 2 μM used in the present study and might explain the low microsomal degradation in RLM.

#### Structure Elucidation of Metabolites and Radiometabolites

Carrier-added (*S*)-[^18^F]**1**, that means the radioligand in presence of (*S*)-**1** (2 μM, unless otherwise stated), was incubated with HLM in PBS at 37°C for 120 min, in presence of NADPH and UDPGA. Both cofactors provide conditions for oxygenation or glucuronidation, respectively, and were used either separately or combined. After termination of the experiment by adding cold acetonitrile, the mixtures were centrifuged and the supernatants investigated by LC-MS/MS, as well as radio-HPLC. The compounds *rac*-**2**, *rac*-**3**, and *rac*-**4** were incubated identically and the prepared samples were analyzed by LC-MS/MS.

##### Detection and structure elucidation of in vitro metabolites of (S)-1 by LC-MS/MS

**FIGURE 3:**
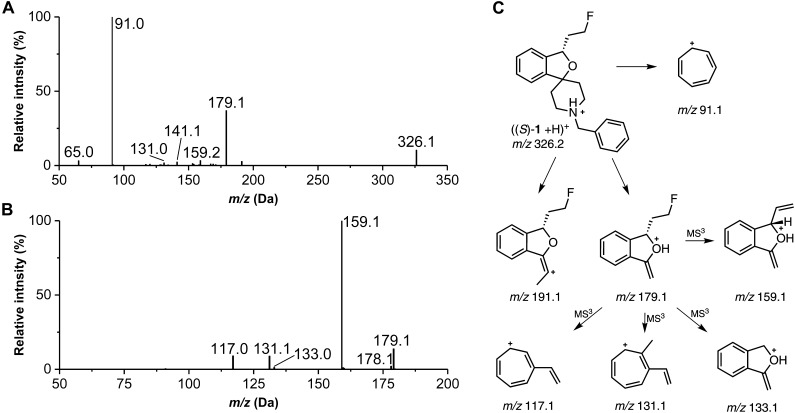
MS/MS data of (S)-**1** (exact mass: 325.18). **(A)** enhanced product ion (EPI) spectrum, precursor ion at m/z 326.2 (CE 40). **(B)** MS3 spectrum of m/z 326.2/179.1 (AF2 0.125). **(C)** Proposed fragmentation pathway for (S)-**1** (in some of the structures of fragment ions positive charges were placed at specific atoms for illustrative purposes).

Prior to LC-MS/MS measurements the parameters for MRM scan mode were optimized using (*S*)-**1** (exact mass: 325.18). In preparation for detailed structural characterization, EPI and MS^3^ measurements were performed for (*S*)-**1** as well as for *rac*-**2**, *rac*-**3**, and *rac*-**4** and fragmentation patterns were interpreted. As shown in Figure 3A, most relevant for (*S*)-**1** was the formation of the tropylium cation [*m/z* 91.1, (C_7_H_7_)^+^]. Consequently, this fragment as well as its derived ions [e.g., *m/z* 107.1, (C_7_H_7_+O)^+^] were used for most of the MRM scans to detect metabolites. Further observed fragment ions observed in EPI or MS^3^ spectra (Figure 3B) were interpreted as shown in Figure 3C.

**FIGURE 4:**
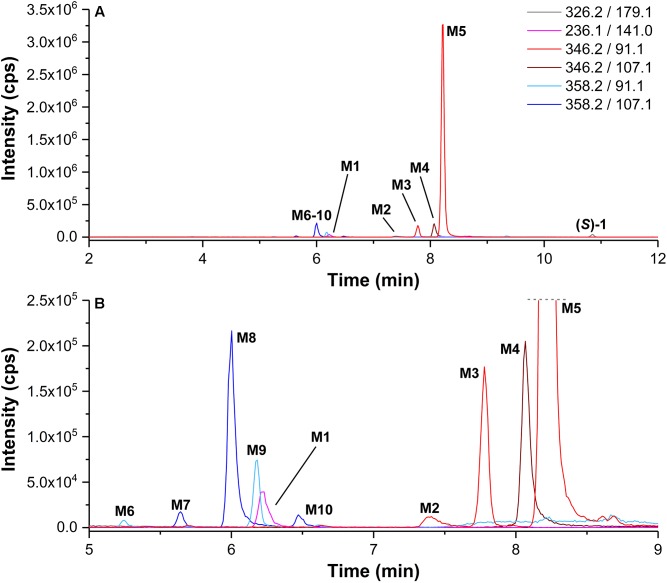
**(A)** *Multiple reaction monitoring* (MRM) chromatograms (Section “LC-MS/MS Analyses”) recorded after incubation of (*S*)-**1** (2 μM) with HLM in presence of NADPH in PBS at 37°C for 120 min (Section “Incubation of (*S*)-[^18^F]**1** and Non-radioactive References for Identification of *in vitro* Metabolites and Radiometabolites”). **(B)** enlarged detail of **A**. MRM transitions as provided in the legend. Data are summarized in Table 1.

For selective detection of metabolites, MRM transitions, which covered products of, e.g., debenzylation, defluorination, single, and multiple oxygenations as well as single and multiple glucuronidation of (*S*)-**1** or its intermediate metabolites, were monitored. After incubation of (*S*)-**1** with HLM in presence of NADPH a series of metabolites (**M1–M10**) could be detected (Figure 4 and Table 1).

**Table 1:** LC-MS/MS data of metabolites (Section “LC-MS/MS Analyses”) detected after incubation of (*S*)-**1** with HLM (NADPH, UDPGA).

Metabolite	t_R_ (min)^a^	MRM transition	EPI fragmentation^b^ (% intensity in brackets)	Metabolic pathway
M1	6.24	236.1/141.0	141.1 (100), 128.0 (64), 154.1 (53), 153.1 (50), 115.1 (29), 129.1 (25), 143.1 (22), 165.0 (10), 117.1 (5), 127.1 (5), 152.0 (5), 155.2 (5), 142.1 (5)	debenzylation
M2	7.38	342.2/91.1	–^c^	*N*-oxidation
M3	7.79	342.2/91.1	91.0 (100), 342.2 (26), 187.1 (7), 185.2 (6), 175.1 (6), 120.1 (6), 205.1 (5), 324.2 (5), 232.1 (4), 167.1 (4), 250.1 (3), 131.1 (3)	hydroxylation (*at piperidine*)
M4	8.07	342.2/107.1	107.0 (100), 236.1 (18), 218.2 (6), 189.1 (4), 169.1 (2), 91.0 (2), 179.1 (2), 107.6 (1), 143.0 (1), 342.1 (1)	hydroxylation (*at 4-phenyl*)
M5	8.22	342.2/91.1	342.2 (100), 195.1 (22), 91.0 (17), 207.1 (2), 205.2 (1), 134.1 (0.9), 250.1 (0.7), 120.0 (0.7), 324.2 (0.4)	hydroxylation (*at fluoroethyl*)
M6	5.24	358.2/91.1	–^c^	di-oxygenation
M7	5.64	358.2/107.1	107.0 (100), 84.0 (91), 139.1 (44), 167.1 (22), 219.2 (11), 122.0 (10), 141.0 (8), 152.2 (8), 134.0 (7), 185.0 (5), 124.1 (5), 112.1 (5), 78.9 (5), 76.9 (4), 129.1 (4)	di-oxygenation (*1x OH at phenyl*)
M8^d^	6.01	358.2/107.1	107.0 (100), 252.1 (81), 205.1 (22), 358.1 (11), 185.1 (6), 234.1 (6), 257.0 (4), 139.1 (4), 83.9 (3), 195.1 (3), 159.1 (3)	di-oxygenation (*1x OH at 4-phenyl*)
M9^d^	6.18	358.2/91.1	91.0 (100), 83.9 (74), 358.2 (35), 147.1 (32), 139.0 (31), 191.1 (20), 149.1 (17), 203.1 (14), 183.1 (12), 256.9 (11), 122.0 (8), 120.0 (8), 155.0 (7), 124.1 (5), 184.9 (5), 141.1 (5), 152.2 (5) 212.2 (5)	di-oxygenation
M10	6.47	358.2/107.1	–^c^	di-oxygenation (*1x OH at phenyl*)
M11	3.72	252.1/141.0	–^c^	debenzylation + oxygenation
M12	3.53	518.2/342.2	342.1 (100), 195.1 (10), 518.1 (8), 207.1 (2), 120.1 (1), 91.0 (0.5)	hydroxylation (*at fluoroethyl*) + glucuronidation
M13, M18, M19, M21^e^	3.00, 4.04, 4.19, 4.45	518.2/342.2	–^c^	oxygenation + glucuronidation
M14, M15, M16, M17, M20^e^	3.22, 3.35, 3.85, 3.98, 4.36	534.2/358.2	–^c^	di-oxygenation + glucuronidation
(*S*)-**1**	10.85	326.2/179.2	91.0 (100), 179.1 (37), 326.1 (10), 159.2 (3), 65.0 (3), 191.1 (3), 141.1 (2), 131.0 (2), 153.1 (1), 167.1 (1) 169.0 (1), 120.1 (1), 154.1 (1), 117.0 (1)	parent

*^a^Retention time; ^b^collision energy (CE) 40, except for **M5** and **M8** (CE 30), further parameters for data acquisition described in Section “LC-MS/MS Analyses”; ^c^not recorded due to low intensity; ^d^EPI spectra were recorded after LC separations at 15°C instead of 40°C to achieve complete separation of **M8** and **M9**; ^e^data have been summarized for reasons of space.*

First, defluorination of (*S*)-**1** was not observed. As also reported for RLM (Holl et al., 2013), (*S*)-**1** underwent debenzylation and metabolite **M1** could be detected by monitoring the MRM transition *m/z* 236.1 (M-CHC_6_H_5_ + H)^+^/141.0. Both the retention time (*t*_R_ = 6.24 min) and the fragmentation pattern obtained by EPI scans matched that of *rac*-**2**. EPI spectra for **M1** and other metabolites are provided in the Supplementary Material.

**FIGURE 5:**
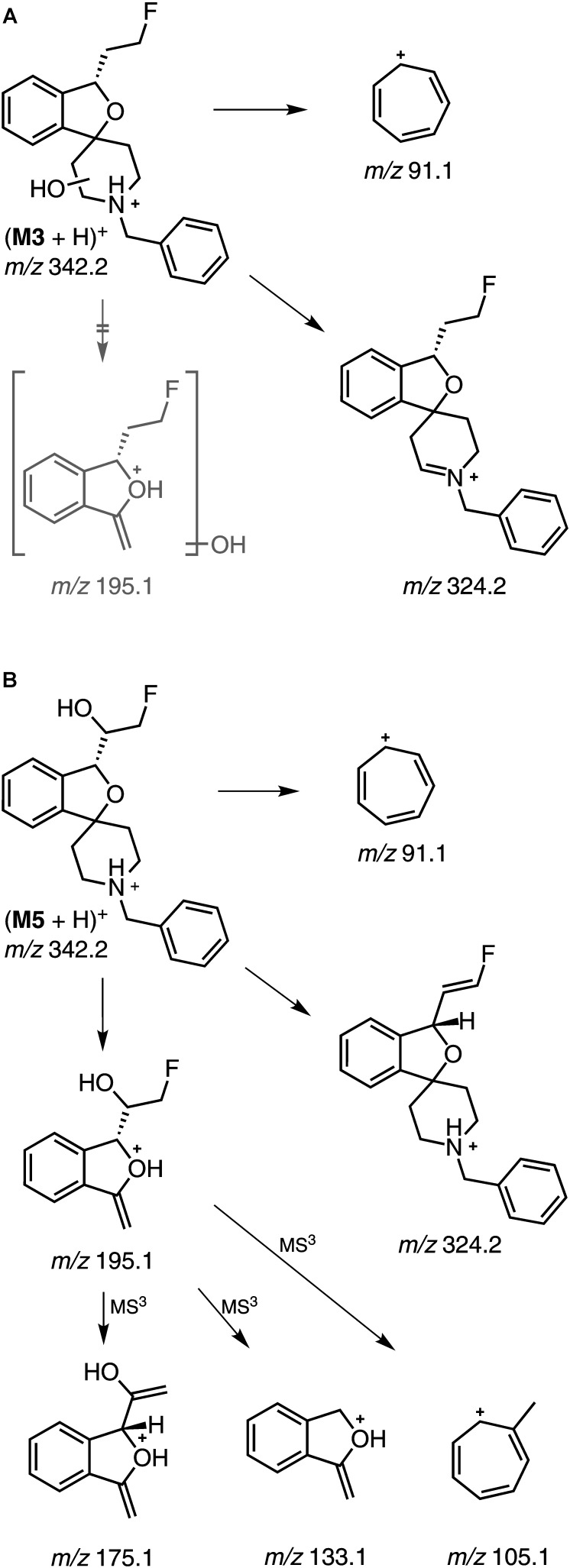
Proposed fragmentation pathways (EPI, MS^3^) for **(A) M3** and **(B) M5** (in some of the structures of fragment ions positive charges were placed at specific atoms for illustrative purposes).

The metabolites **M2**–**M5** were formed by single oxygenation of (*S*)-**1**. Two of them, **M2** and **M4**, could be characterized by comparison with synthesized references. The retention time of **M2** (*t*_R_ = 7.38 min) matched that of the *N*-oxide *rac*-**4** and the fragmentation pattern for *m/z* 342.2 (M+O +H)^+^ appeared similar. However, **M2** was most likely not a product of CYP-mediated oxidation, since it was detected also in NADPH-free incubations with comparable low intensity. **M4** (*t*_R_ = 8.07 min) was identified as an hydroxylation product of (*S*)-**1**, bearing the hydroxyl function at the phenyl ring of the benzyl substituent, proven by an MRM transition of *m/z* 342.2 (M+O +H)^+^/107.1 (C_7_H_7_+O)^+^. Both the retention time and the fragmentation pattern were highly similar to that of *rac*-**3**, which indicates that **M4** was hydroxylated at the *para* position of the phenyl ring. In contrast, **M3** could be detected by recording an MRM transition of *m/z* 342.2 (M+O +H)^+^/91.1 (C_7_H_7_)^+^. In this case, a hydroxylation at the benzyl group could be excluded, due to the occurrence of the tropylium cation [(C_7_H_7_)^+^] as for (*S*)-**1**. In the EPI spectrum, the fragment ion *m/z* 324.2 resulted from a loss of water (*m/z* -18) as one can expect as a result of a hydroxylation at the piperidine moiety. This interpretation was underpinned by the absence of a corresponding oxygenated methylene-dihydroisobenzofuranium fragment ion (*m/z* 195.1) (Figure 5A). In contrast, for **M5** the fragment ion of *m/z* 195.1 provided evidence for a hydroxylation at the fluoroethyl-dihydroisobenzofuran moiety of the molecule (Figure 5B). Subsequent fragmentation in MS^3^ experiments further revealed a hydroxylation at the fluoroethyl side chain, as also substantiated by detected elimination of water (*m/z* -18). Beside MS^3^ data, in particular the fragment ion *m/z* 175.1, suggests that hydroxylation took place at the carbon atom next to the chiral center of the molecule, as it has been discussed in literature (Holl et al., 2013). As reported, after incubation with RLM, a hydroxylation at the fluoroethyl side chain was observed only when (*S*)-**1** but not (*R*)-**1** was incubated, which indicated that a reaction at the carbon atom closer to the chiral center appears to be more likely.

**FIGURE 6:**
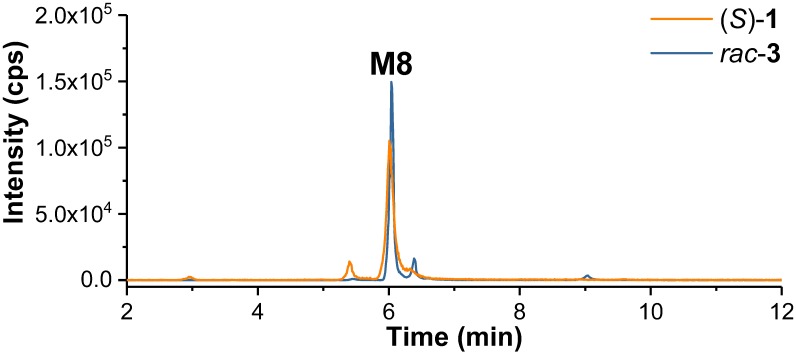
*Multiple reaction monitoring* (MRM) chromatograms (m/z 358.2/107.1) (Section “LC-MS/MS Analyses,” LC temperature 15°C instead of 40°C) recorded after incubation of (*S*)-**1** and *rac*-**3** (intensity reduced by factor 15) with HLM in presence of NADPH (Section “Incubation of (*S*)-[^18^F]**1** and Non-radioactive References for Identification of *in vitro* Metabolites and Radiometabolites”).

As shown in Figure 4, also products from di-oxygenations (**M6–M10**) were found. Since they were detected by one of the MRM transitions either *m/z* 358.2 (M+2O +H)^+^/91.1 (C_7_H_7_)^+^ or *m/z* 358.2 (M+2O +H)^+^/107.1 (C_7_H_7_+O)^+^, they could be divided in those with absent (**M6**, **M9**) and those with a single hydroxyl function (**M7**, **M8**, **M10**) at the phenyl ring. HLM incubation of the *para*-hydroxy-phenyl derivative *rac*-**3** [instead of (*S*)-**1**] resulted in the formation of a di-oxygenated metabolite that matched **M8** with regard to its retention time (Figure 6). The EPI spectrum of **M8** showed a loss of water (*m/z* -18) which provided an indication of a hydroxyl function either at the piperidine moiety or the fluoroethyl chain (Figure 10).

**FIGURE 7:**
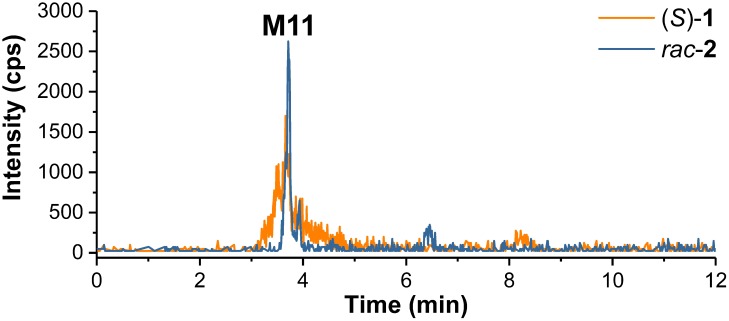
*Multiple reaction monitoring* (MRM) chromatograms (m/z 252.1/141.0) (Section “LC-MS/MS Analyses,” LC temperature 15°C instead of 40°C) recorded after incubation of (*S*)-**1** and *rac***-2** with HLM in presence of NADPH (Section “Incubation of (*S*)-[^18^F]**1** and Non-radioactive References for Identification of *in vitro* Metabolites and Radiometabolites”).

The possible metabolism by debenzylation and hydroxylation was studied in detail, since it has been reported for incubation with RLM (Holl et al., 2013). For that purpose, *rac*-**2** instead of (*S*)-**1** was incubated with HLM in presence of NADPH. By recording the MRM transition *m/z* 252.1/141.0 the minor metabolite **M11** (*t*_R_ = 3.72 min) was detected after incubation of both (*S*)-**1** and *rac*-**2** (Figure 7). However, due to very low signal intensities no further characterization was possible.

The *in vitro* metabolites of (*S*)-**1** detected in the current study after incubation with HLM correspond, to a large extent, to those reported from RLM incubations (Holl et al., 2013). Debenzylation (**M1**) and mono-oxygenations (**M2**–**M5**) were observed for both cases, even though in different proportions. Further, 5 and 3 di-oxygenated products were detectable after incubation with HLM and RLM, respectively, which might be a result of different MS detectors and methods used in both studies. However, a twofold hydroxylation at the phenyl moiety reported for RLM was not found for HLM.

**FIGURE 8:**
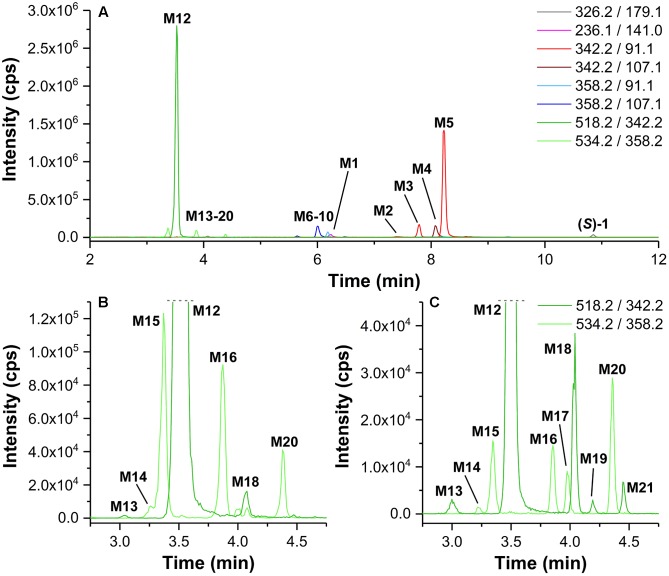
**(A)** *Multiple reaction monitoring* (MRM) chromatograms (Section “LC-MS/MS Analyses”) recorded after incubation of (*S*)-**1** (2 μM) with HLM in presence of NADPH and UDPGA in PBS at 37°C for 120 min (Section “Incubation of (*S*)-[^18^F]**1** and Non-radioactive References for Identification of *in vitro* Metabolites and Radiometabolites”). **(B)** enlarged detail of **A**, showing MRM chromatograms for glucuronide conjugates of mono- and di-oxygenated metabolites. **(C)** detail according to **B**, but after incubation of (*S*)-**1** at 200 μM. MRM transitions as provided in the legends. Data are summarized in Table 1.

Samples from incubations of (*S*)-**1** with HLM were investigated regarding the formation of conjugates with glucuronic acid by monitoring MRM transitions corresponding to a neutral loss of *m/z* 176.0 (C_6_H_8_O_6_) as characteristic for glucuronides in positive-ion mode (Levsen et al., 2005). Only after incubation in presence of both cofactors NADPH and UDPGA glucuronide formation of (*S*)-**1** was observed. Thus, not (*S*)-**1** but its intermediate metabolites, previously formed by oxygenations, were glucuronidated. As shown in Figure 8A, one main glucuronide (**M12**, *t*_R_ = 3.53 min), formed after mono-oxygenation was detected by monitoring the MRM transition 518.2 (M+O+C_6_H_8_O_6_ +H)^+^/342.1 (M+O +H)^+^. In addition, glucuronide conjugates of previously formed di-oxygenation products were detected, but with low signal intensities (Figure 8A,B). After incubation with (*S*)-**1** at a concentration of 200 μM instead of 2 μM (Figure 8C) 5 mono-oxygenated glucuronides (**M12**, **M13**, **M18**, **M20**, **M21**) and 5 di-oxygenated glucuronides (**M14**-**M17**, **M20**) were found.

**FIGURE 9:**
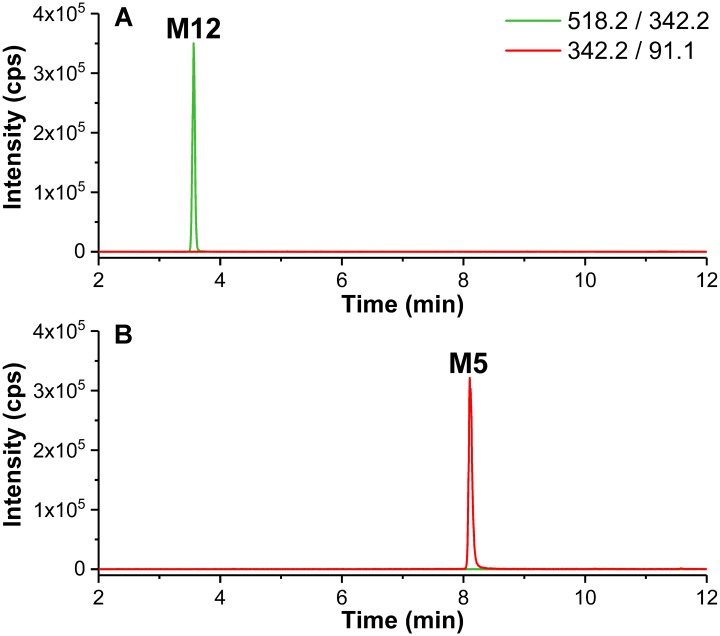
*Multiple reaction monitoring* (MRM) chromatograms (Section “LC-MS/MS Analyses”) recorded before **(A)** and after **(B)** incubation of **M12** with *β*-glucuronidase (*Helix pomatia* type H-3) (Section “*β*-Glucuronidase Cleavage of Microsomal-Formed Metabolite **M12**”). MRM transitions as provided in the legend.

For the main glucuronide **M12**, collision-induced fragment ions at *m/z* 342.1, 324.1 and in particular *m/z* 195.1 correspond to those found for the hydroxyl-fluoroethyl metabolite **M5**, which provides a clear indication that it serves as an intermediate for subsequent glucuronidation. For further validation, the glucuronide cleavage was studied for **M12**. In brief, a solution of **M12**, obtained from HLM incubations and subsequent HPLC separation, was stirred at 37°C with *β*-glucuronidase (*Helix pomatia* type H-3) in acetate buffer (Yilmazer et al., 2001; Xu et al., 2002) and samples were inspected by measuring appropriate MRM transitions. During incubation with *β*-glucuronidase **M12** was cleaved completely whereas **M5** was the only product observed (Figure 9), also proven by comparison with LC-MS/MS data from HLM incubation.

**FIGURE 10:**
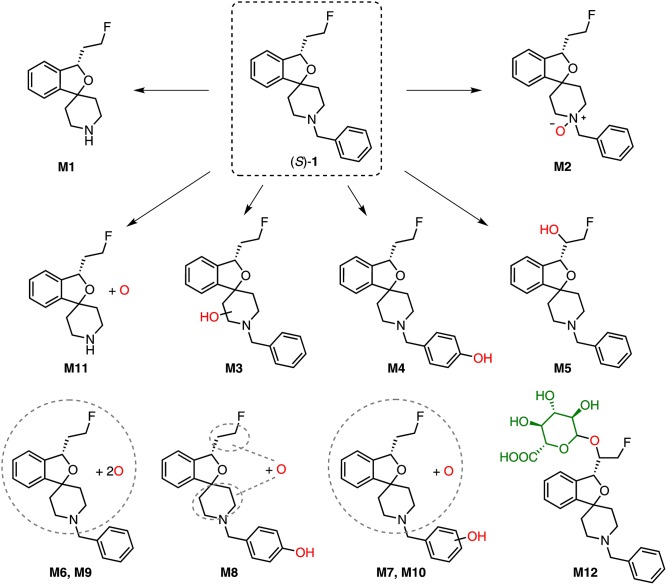
Structures of *in vitro* metabolites detected after incubation of (*S*)-**1** with HLM (NADPH, UDPGA).

##### Identification of in vitro radiometabolites of (S)-[^18^F]1

**FIGURE 11:**
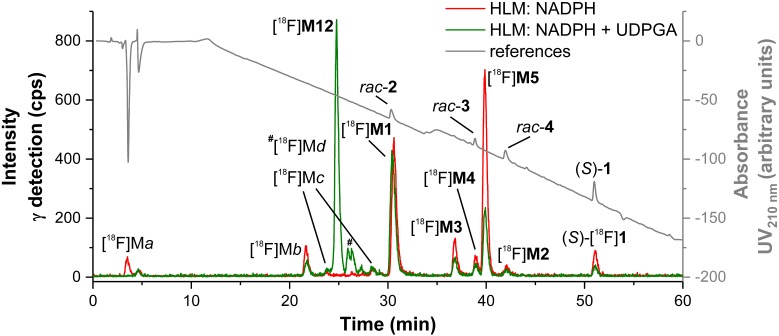
Radio-HPLC chromatograms (system I, Section “Radio-HPLC”) recorded after incubation of (*S*)-[^18^F]**1** (carrier-added, (*S*)-**1**, 2 μM) with HLM (NADPH and UDPGA as stated in the legend) (Section “Incubation of (*S*)-[^18^F]**1** and Non-radioactive References for Identification of *in vitro* Metabolites and Radiometabolites”) combined with an UV-HPLC chromatogram (210 nm) of a mixture of references.

**FIGURE 12:**
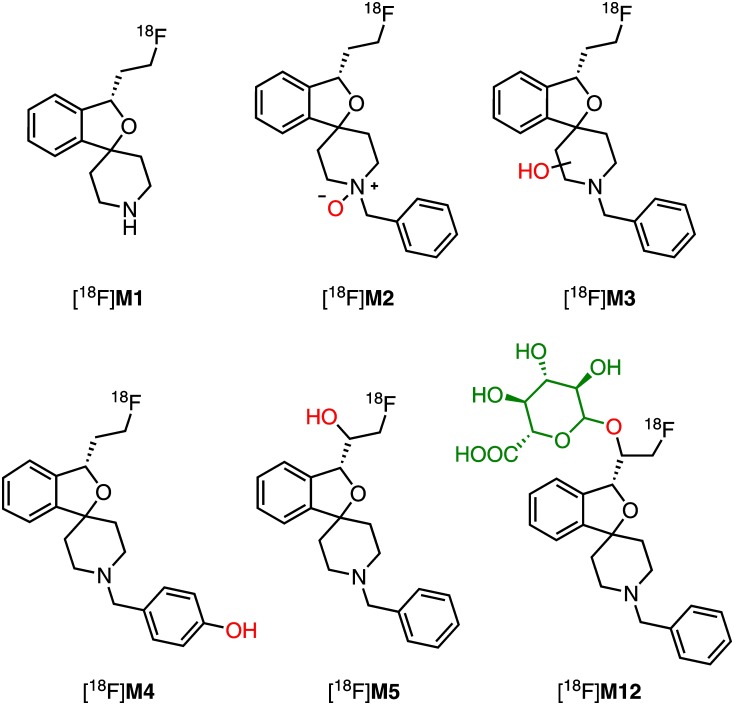
Overview of structures of identified *in vitro* radiometabolites found after in incubation of (*S*)-[^18^F]**1** with HLM (NADPH, UDPGA).

After HLM incubation in presence of NADPH a series of radiometabolites was detected by HPLC with a radioactivity flow detector (Figure 11). Incubations with both NADPH and UDPGA resulted in further products, due to glucuronide conjugation. Generally, patterns of radiometabolites resulting from (*S*)-[^18^F]**1** largely matched those of metabolites of (*S*)-**1** in LC-MS/MS (MRM) chromatograms. First assignments were done by comparative measurements using *rac*-**2**, *rac*-**3**, *rac*-**4** with UV monitoring at 210 nm. Thus, [^18^F]**M1**, [^18^F]**M2**, and [^18^F]**M4** could be characterized as products of debenzylation, *N*-oxidation, and hydroxylation at the *para* position of the phenyl ring, due to their co-elution with the corresponding non-radioactive references (Figure 11). It is interesting to note that the *N*-oxide [^18^F]**M2** eluted later than [^18^F]**M3**-[^18^F]**M5**, which is in contrast to the elution order observed in LC-MS/MS. However, by comparison with data from LC-MS/MS, [^18^F]**M3** and [^18^F]**M5** could clearly be identified as products of mono-hydroxylation at the piperidine moiety and the fluoroethyl side chain, respectively. The same applies to the UDPGA-dependently formed radiometabolite [^18^F]**M12**, which was deduced as formed by a hydroxylation at the fluoroethyl side chain of (*S*)-[^18^F]**1** and subsequent glucuronidation, as demonstrated for the mainly formed non-radioactive glucuronic acid conjugate **M12** (Section “Detection and structure elucidation of *in vitro* metabolites of (*S*)-**1** by LC-MS/MS”). Further, minor ^18^F-bearing glucuronides were detected ([^18^F]M*d*) but could only tentatively be assigned to glucuronides formed after previous mono- or di-oxygenation, as numerous of such corresponding non-radioactive products were found by LC-MS/MS (Figure 8). The molecular identity of further radiometabolites ([^18^F]M*a*-[^18^F]M*c*) could not be elucidated. Thus, to interpret [^18^F]M*b* as a product of debenzylation and additional oxygenation or [^18^F]M*c* as multiple oxygenation products remains speculative, although those transformations were demonstrated for (*S*)-**1** (Section “Detection and structure elucidation of *in vitro* metabolites of (*S*)-**1** by LC-MS/MS”). Structures of identified *in vitro* radiometabolites of (*S*)-[^18^F]**1** are summarized in Figure 12.

### Investigation of the Metabolism of (*S*)-[^18^F]1 in Human

After administration of 244.6-290.4 MBq (mean: 265.5 MBq) (*S*)-[^18^F]**1** to eight healthy controls, plasma and urine samples were taken at 10, 20, and 30 min post injection and measured by radio-HPLC as described for the *in vitro* investigations in Section “Identification of *in vitro* radiometabolites of (*S*)-[^18^F]**1**.” Plasma samples were prepared by adding cold acetonitrile followed by centrifugation and evaporation of the supernatant. Two different procedures (method A and B), using different volumes of plasma and solvent, including a second extraction step of the formed residue, were established. For both methods, the recovery of activity was in the range of 92–97%.

Urine samples, taken after 90 or 120 min post injection were measured by radio-HPLC without further preparation.

#### Metabolic Stability in Humans

**FIGURE 13:**
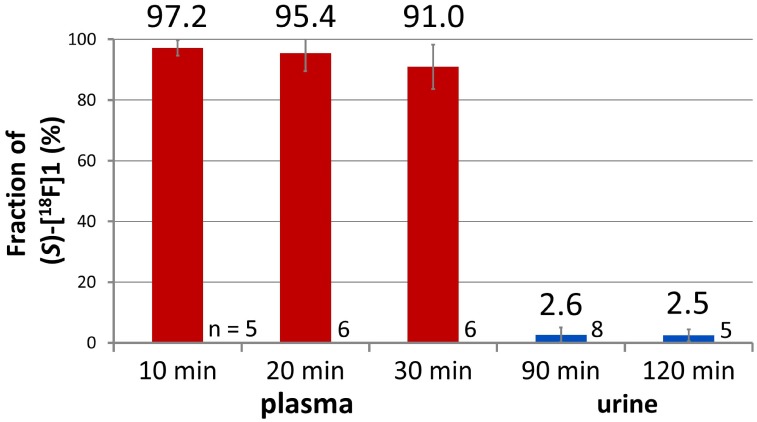
Fractions of intact (*S*)-[^18^F]**1** in plasma and urine (Section “Investigation of the Metabolism of (*S*)-[^18^F]**1** in Humans”) determined by radio-HPLC (system I, Section “Radio-HPLC”), mean values ± SD (error bars).

Samples of plasma showed a high fraction of intact (*S*)-[^18^F]**1** (Figure 13). At 10, 20, and 30 min post injection (*S*)-[^18^F]**1** still represented 97.2 ± 2.6% (mean ± SD), 95.4 ± 5.9%, and 91.0 ± 7.3% of the total plasma activity. In urine, at 90 and 120 min post injection, (*S*)-[^18^F]**1** represented 0.0–7.9% of the total activity due to the high fraction of excreted radiometabolites.

Metabolism rates have been reported for *rac*-[^18^F]**1** or (*S*)-[^18^F]**1** in mice, pigs, and monkeys. In mouse plasma the fraction of unchanged *rac*-[^18^F]**1** was 89 ± 3% at 30 min post injection (Wiese et al., 2016). For (*S*)-[^18^F]**1** it was shown that in plasma of piglets 37% of the radioligand remained unchanged at 30 min post injection (Brust et al., 2014a). In rhesus monkeys (*S*)-[^18^F]**1** still represented 50% of the total activity in plasma at the same time point (Baum et al., 2017), which is less than found for *rac*-[^18^F]**1** in mice (89%). Surprisingly, the estimated value for (*S*)-[^18^F]**1** in human plasma (91.0% at 30 min post injection) is in considerable accordance with published *in vivo* data from mice.

The obtained *in vitro* data (Section “Time- and Concentration-Dependent Microsomal Transformation”) could not predict the levels of (*S*)-[^18^F]**1** in human plasma, due to further metabolic pathways beside CYP-mediated degradation, in particular conjugation with glucuronic acid (Section “Characterization of Radiometabolites Formed in Humans”) and resulting excretion.

#### Characterization of Radiometabolites Formed in Humans

**FIGURE 14:**
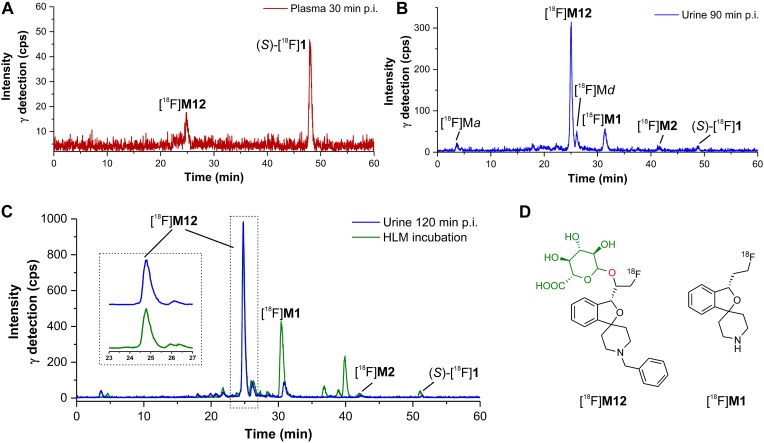
Identification of radiometabolites of (*S*)-[^18^F]**1** formed in humans (Sections “Investigation of the Metabolism of (*S*)-[^18^F]**1** in Humans” and “Incubation of (*S*)-[^18^F]**1** and Non-radioactive References for Identification of *in vitro* Metabolites and Radiometabolites”). **(A)** radio-HPLC chromatogram from plasma (30 min post injection). **(B)** radio-HPLC chromatogram from urine (90 min post injection). **(C)** comparison of radio-HPLC chromatograms from urine (120 min post injection) and HLM incubation (NADPH, UDPGA), including enlarged section. **(D)** structures of identified main radiometabolites formed in humans ([^18^F]**M12**: plasma and urine, [^18^F]**M1**: urine). All chromatograms were recorded using radio-HPLC system I (Section “Radio-HPLC”).

In Figure 14 representative radio-HPLC chromatograms from plasma (**A**) and urine (**B**) samples are shown, obtained after administration of (*S*)-[^18^F]**1**. For characterization of radiometabolites the chromatograms from urine and HLM incubations (Section “Detection and structure elucidation of *in vitro* metabolites of (*S*)-**1** by LC-MS/MS”) were compared (Figure 14C). Several previously characterized *in vitro* radiometabolites are also formed *in vivo* in human. The main radiometabolite detected in urine and plasma was identified as the glucuronide conjugate [^18^F]**M12**, which was formed after hydroxylation at the fluoroethyl side chain (Figure 14D). In plasma [^18^F]**M12** was the only radiometabolite detected and increased over time. As found after incubations with HLM, debenzylation and to a very low extent also *N*-oxidation was observed resulting in [^18^F]**M1** and [^18^F]**M2**, respectively. Further *in vivo* radiometabolites could not be identified with certainty, although found *in vitro*. For example, the fast eluting [^18^F]M*a* most likely refers to [^18^F]fluoride, whereas [^18^F]M*d* might result from further glucuronide conjugates as discussed in Section “Identification of *in vitro* radiometabolites of (*S*)-[^18^F]**1**.”

Deduced from the retention behaviors in the radio-HPLC, no radiometabolite appeared to have a higher lipophilicity than (*S*)-[^18^F]**1**. Taking into account that in mouse brain 98% of *rac*-[^18^F]**1** remained unchanged at 60 min post injection (Wiese et al., 2016), the absence of radiometabolites in human brain is highly likely as well.

## Conclusion

As demonstrated, radiometabolites of (*S*)-[^18^F]**1** formed *in vivo* in humans could be characterized by means of *in vitro* investigations and LC-MS/MS. For that purpose HLM were used in presence of NADPH and UDPGA to generate metabolites of (*S*)-**1** as well as the corresponding radiometabolites of (*S*)-[^18^F]**1**, which revealed to be relevant *in vivo*. Investigations by LC-MS/MS and comparison with obtained radio-HPLC data showed that debenzylation, hydroxylation at the fluoroethyl side chain, and a subsequent glucuronidation were predominant for metabolic degradation *in vitro*. Further, minor oxygenated metabolites were detected and characterized. Defluorination, which is a critical aspect of a radioligand and leads to non-specific accumulation of radioactivity in bone tissue resulting from ^18^F-fluoride, was not observed. In human plasma unchanged (*S*)-[^18^F]**1** represented 91% of the total activity at 30 min post injection. Based on results obtained *in vitro*, formed radiometabolites could be characterized. Thus, hydroxylation at the fluoroethyl side chain of (*S*)-[^18^F]**1** and subsequent conjugation with glucuronic acid ([^18^F]**M12**) occurred as the main metabolic pathway in humans. Besides, debenzylation of the molecule was observed ([^18^F]**M1**). Our metabolic study, in particular the high fractions of unchanged radioligand in plasma, confirms the suitability of (*S*)-[^18^F]fluspidine ((*S*)-[^18^F]**1**) as PET radioligand for sigma-1 receptor imaging.

## Data Availability

The raw data supporting the conclusions of this manuscript will be made available by the authors, without undue reservation, to any qualified researcher.

## Ethics Statement

The use of (*S*)-[^18^F]fluspidine ((*S*)-[^18^F]**1**) for human application was authorized by the competent authorities in Germany, the Federal Institute for Drugs and Medical Devices (Bundesamt für Arzneimittel und Medizinprodukte, BfArM) and the Federal Office for Radiation Protection (Bundesamt für Strahlenschutz, BfS) as well as by the local ethics committee. The study was conducted in accordance with the Declaration of Helsinki. Informed consent was obtained from all healthy volunteers (age ≥ 18). All investigations were conducted in the framework of an approved and registered clinical study EudraCT-Nr.: 2014- 005427-27.

## Author Contributions

F-AL, SF, AH, WD-C, DS, MP, JS, BW, and PB conceived and designed the experiments. F-AL, SF, RH, MP, and FZ conducted the experiments. F-AL, SF, RH, and MP analyzed the data. F-AL, SF, RH, AH, DS, MP, PMM, SH, G-AB, FZ, JS, BW, OS, and PB wrote or contributed to the writing of the manuscript.

## Conflict of Interest Statement

AH is employed by company ABX advanced biochemical compounds GmbH. The remaining authors declare that the research was conducted in the absence of any commercial or financial relationships that could be construed as a potential conflict of interest.
