# Supplementary material for: In vitro and in vivo Human Metabolism of (S)-[18F]Fluspidine – A Radioligand for Imaging σ1 Receptors With Positron Emission Tomography (PET)
Source: Front Pharmacol. 2019 Jun 13;10:534. doi: 10.3389/fphar.2019.00534 (PMC6585474; doi:10.3389/fphar.2019.00534)
Supplement: Supplementary file 1 [file Data_Sheet_1.docx]

In vitro and in vivo Human Metabolism of (S)-[^18^F]Fluspidine – a Radioligand for Imaging σ_1_ Receptors with Positron Emission Tomography (PET)

Supplementary Material

## EPI spectra of detected *in vitro* Metabolites

The figures show *enhanced product ion* spectra (EPI) of *in vitro* metabolites of (*S*)-**1** corresponding to the data summarized in **Table 1** in the article.


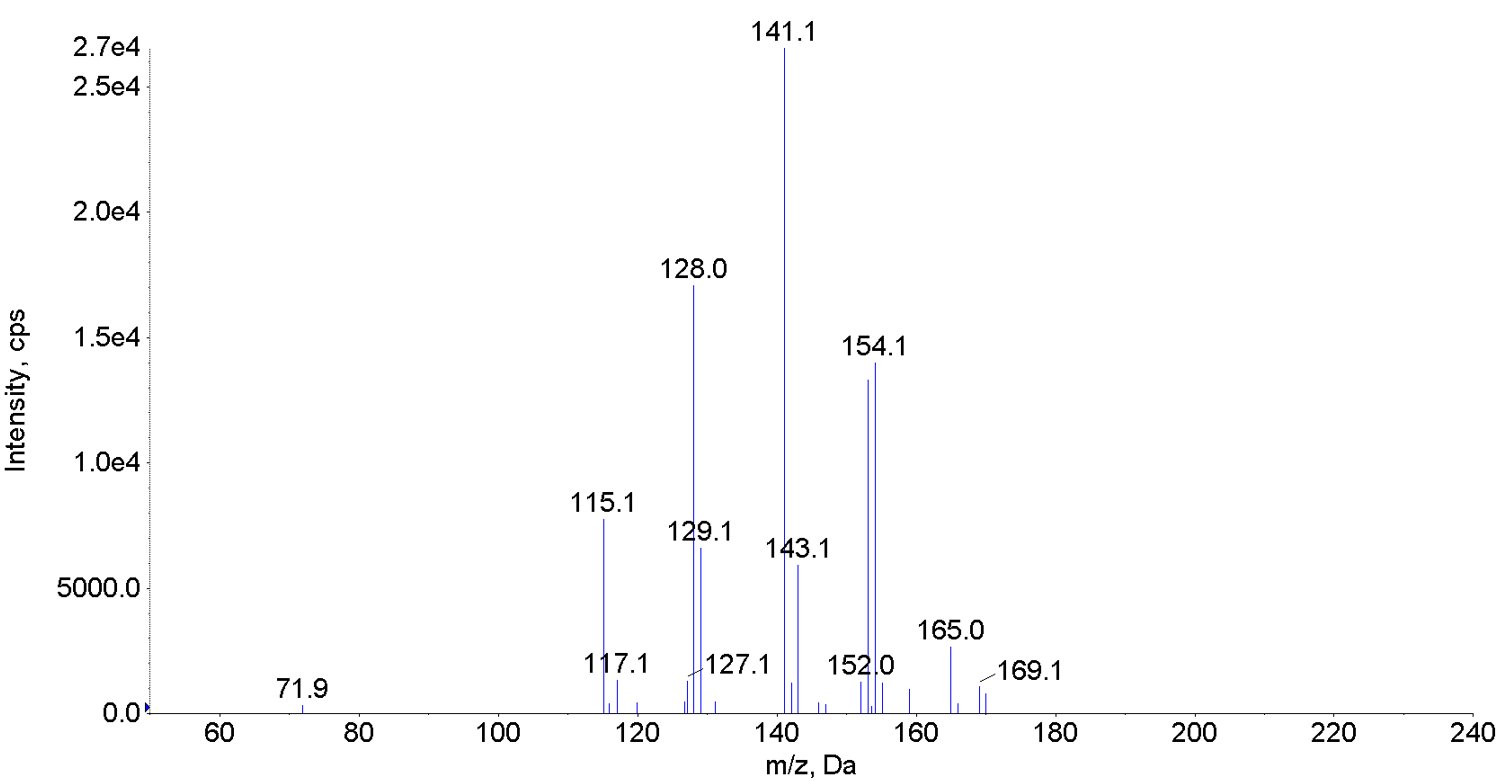


**Supplementary Figure 1.** EPI spectrum of **M1**, precursor ion at *m/z* 326.2 (collision energy (CE) 40).


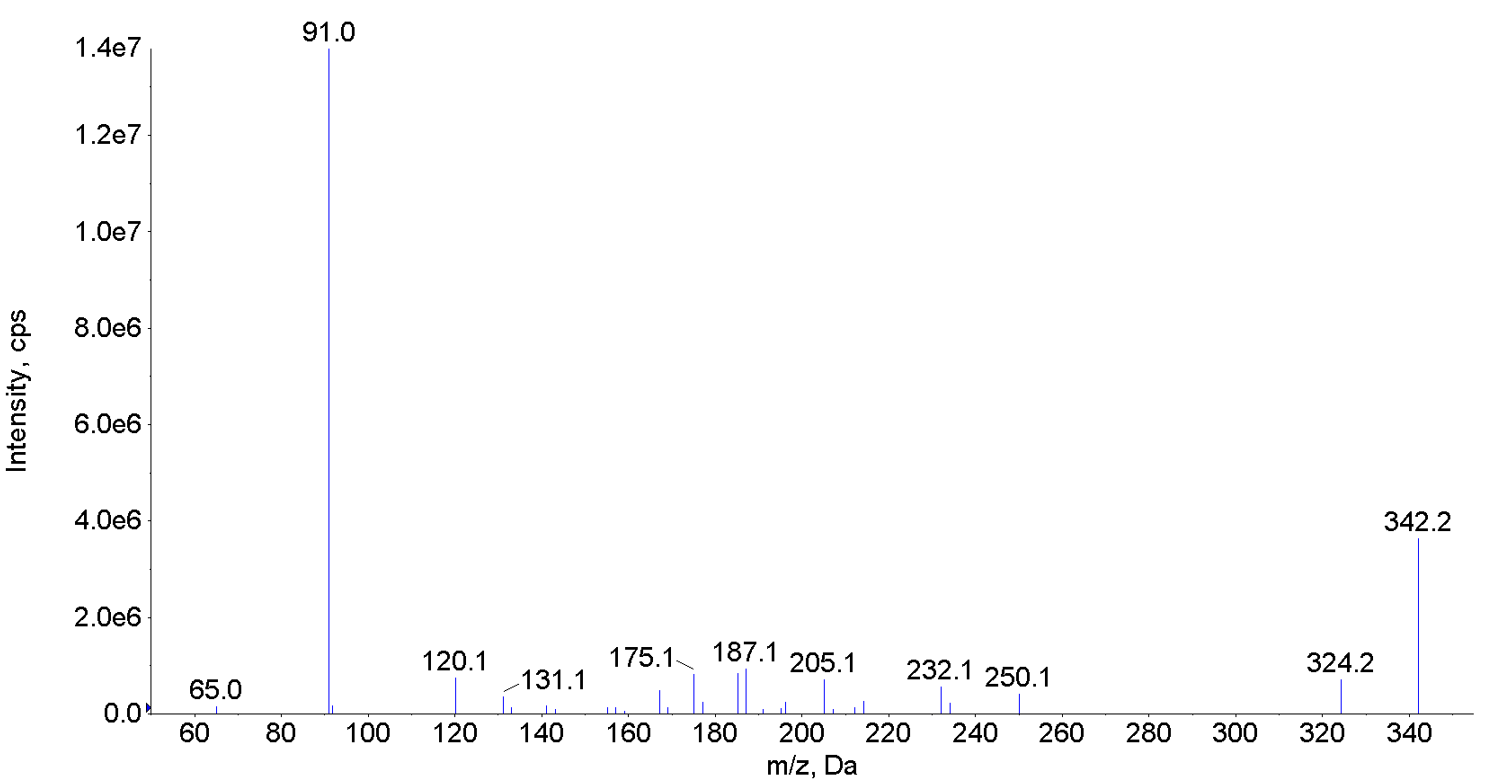


**Supplementary Figure 2.** EPI spectrum of **M3**, precursor ion at *m/z* 342.2 (CE 30).


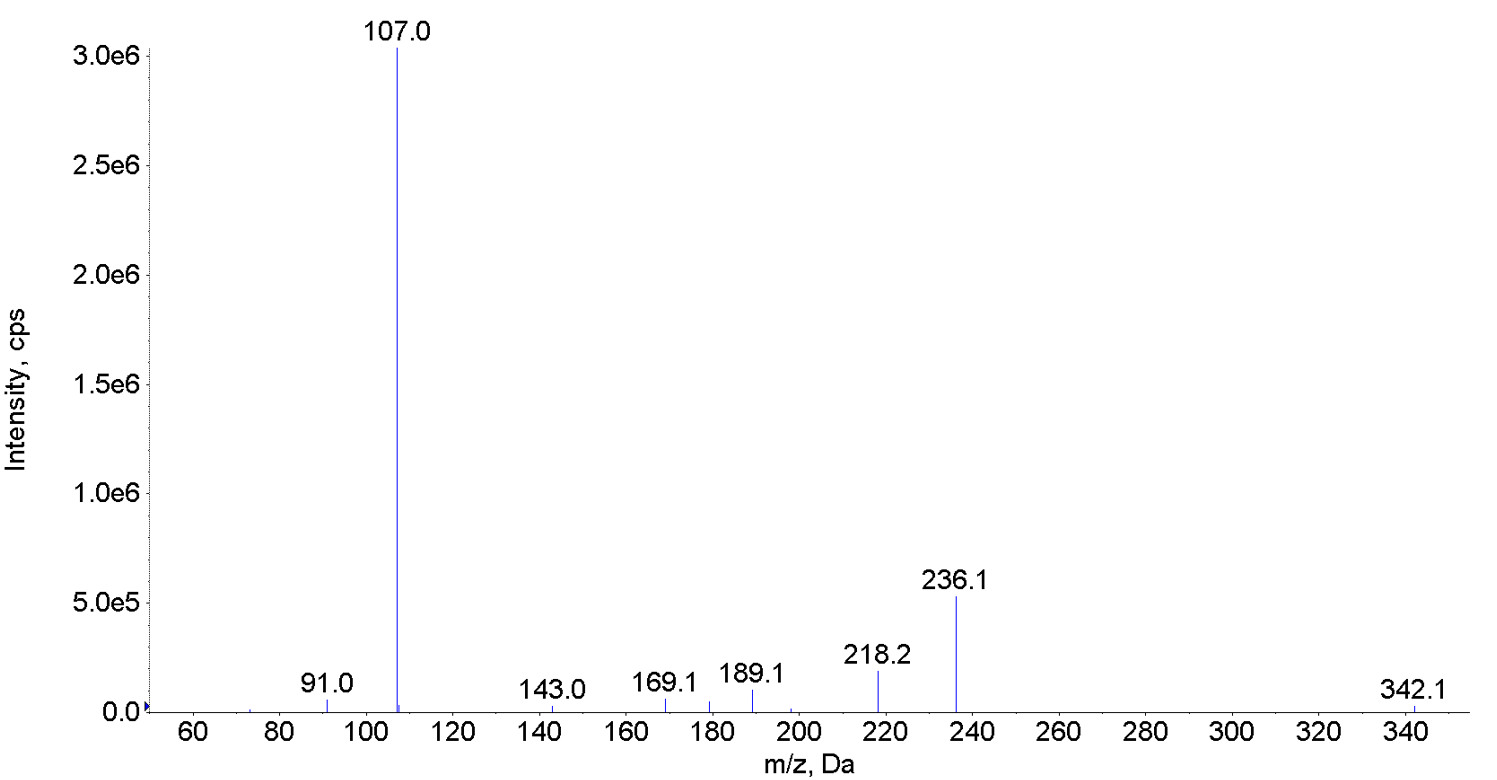


**Supplementary Figure 3.** EPI spectrum of **M4**, precursor ion at *m/z* 342.2 (CE 40).


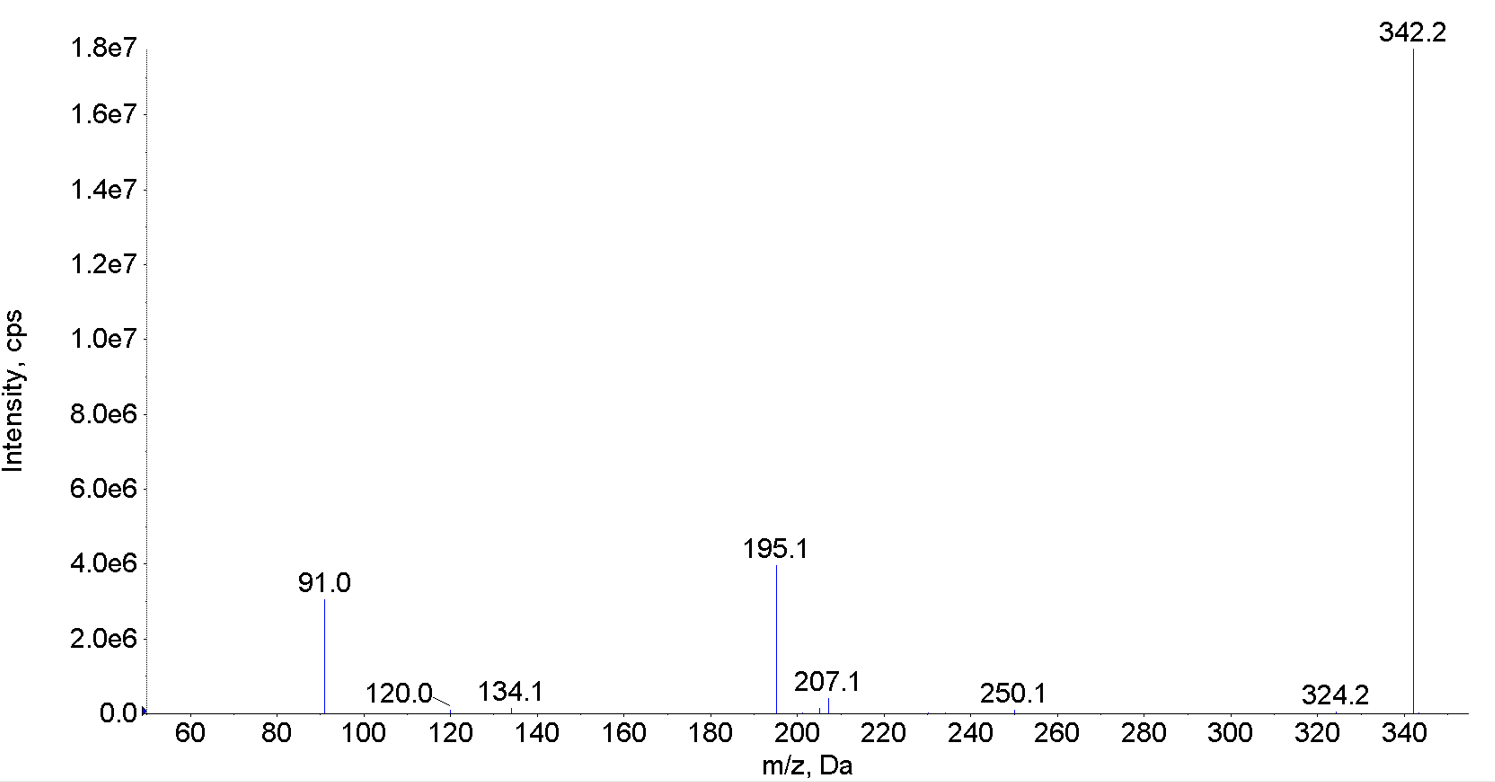


**Supplementary Figure 4.** EPI spectrum of **M5**, precursor ion at *m/z* 342.2 (CE 30).


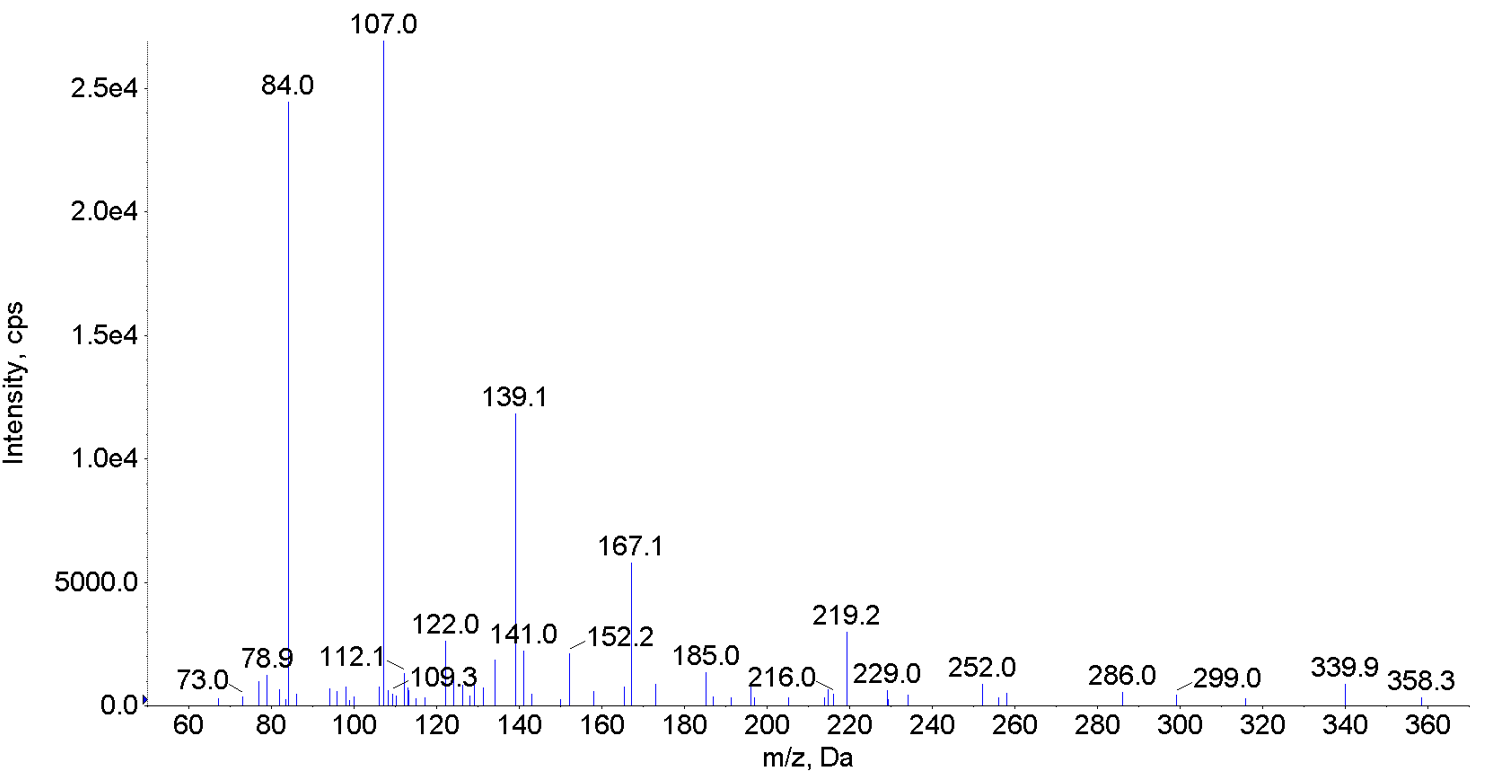


**Supplementary Figure 5.** EPI spectrum of **M7**, precursor ion at *m/z* 358.2 (CE 40).


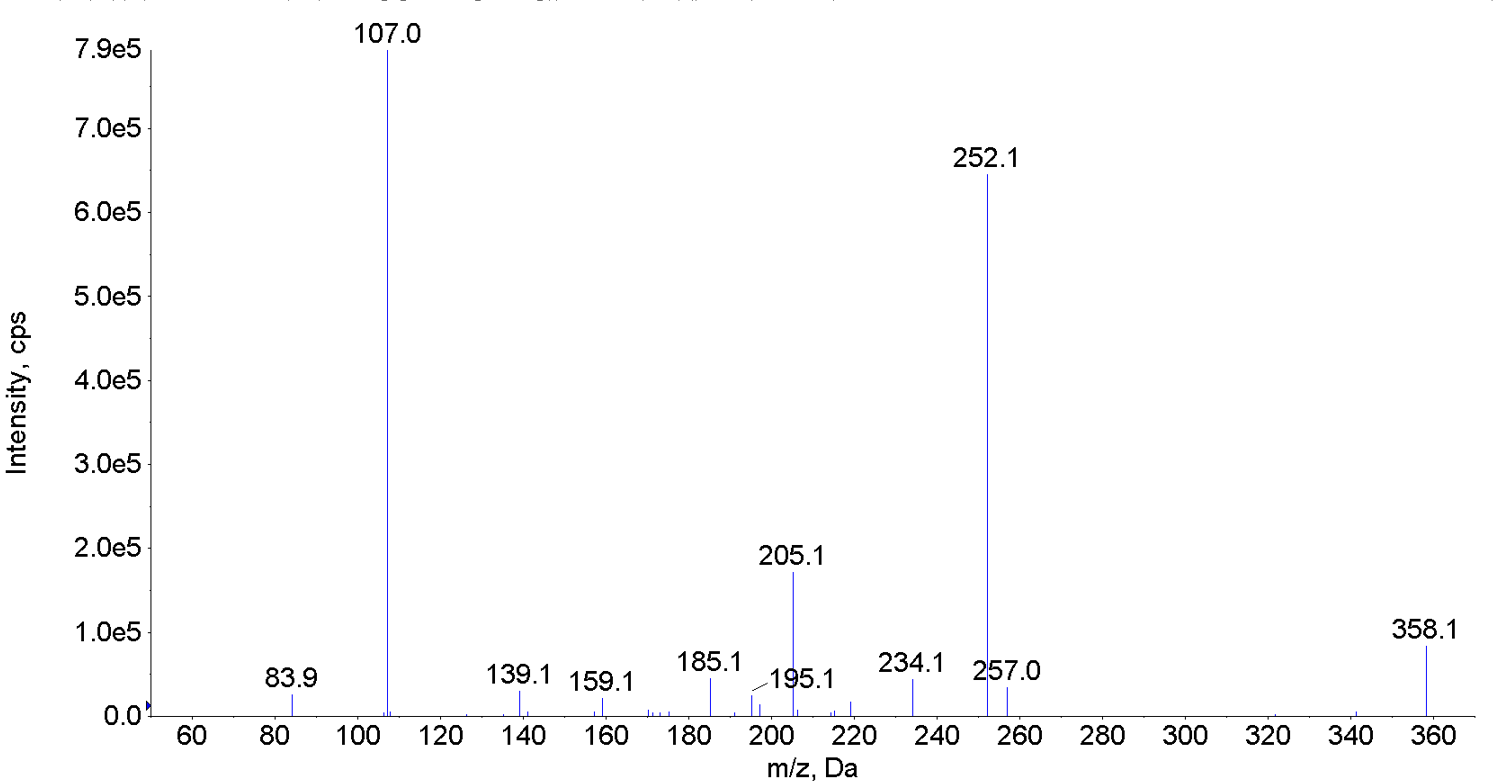


**Supplementary Figure 6.** EPI spectrum of **M8**, precursor ion at *m/z* 358.2 (CE 30).


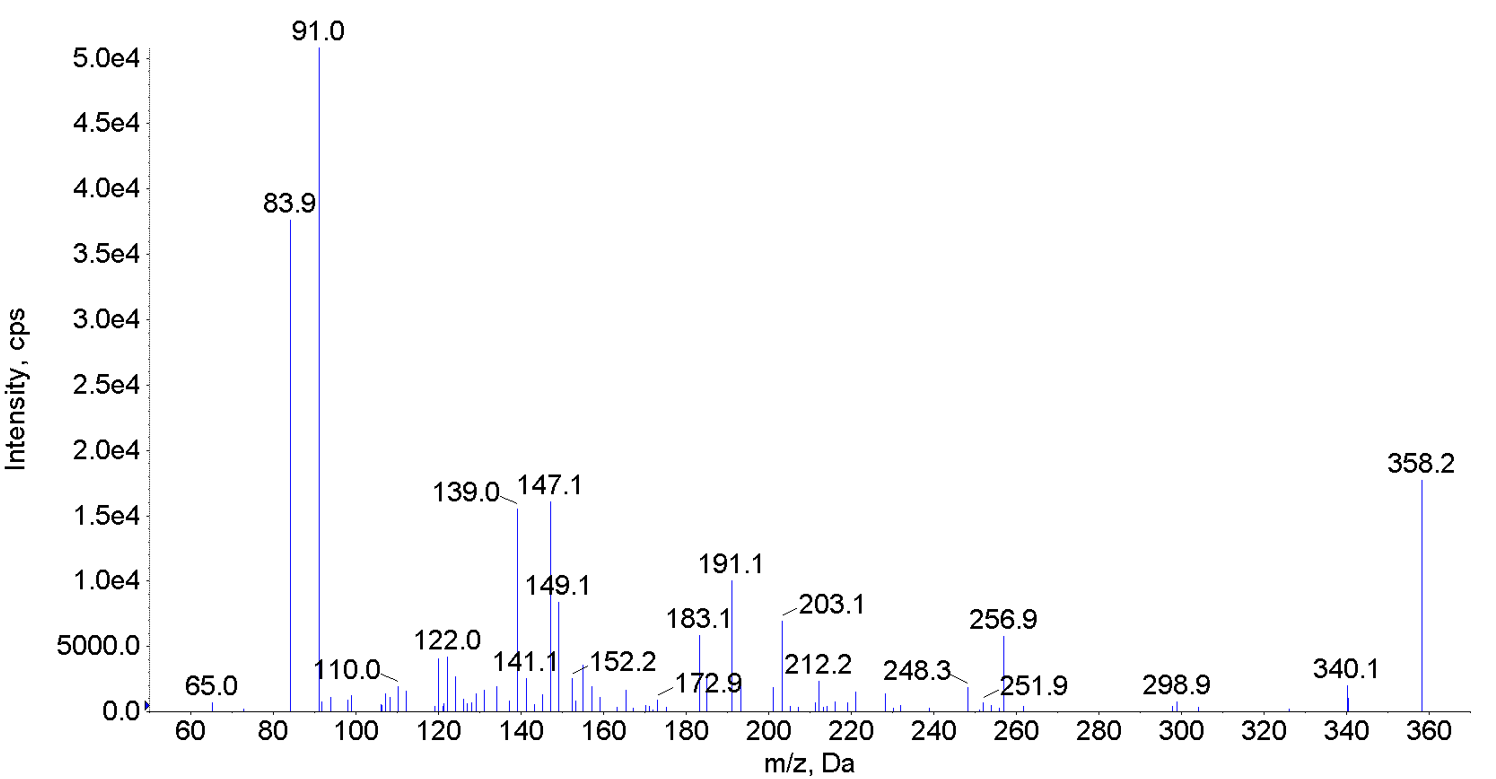


**Supplementary Figure 7.** EPI spectrum of **M9**, precursor ion at *m/z* 358.2 (CE 40).


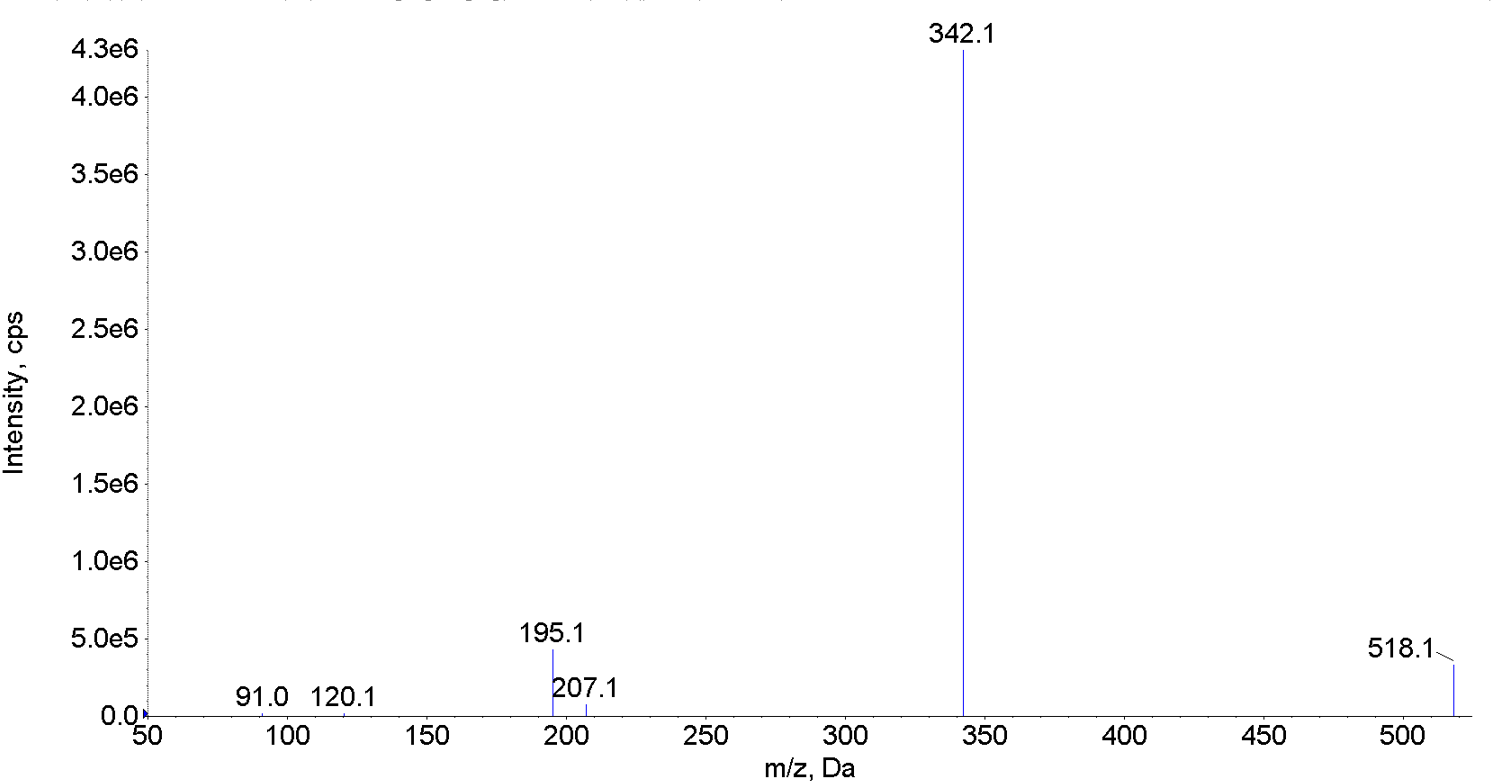


**Supplementary Figure 8.** EPI spectrum of **M12**, precursor ion at *m/z* 518.2 (CE 40).

## Selected MS^3^ spectra recorded for *in* *vitro* Metabolites


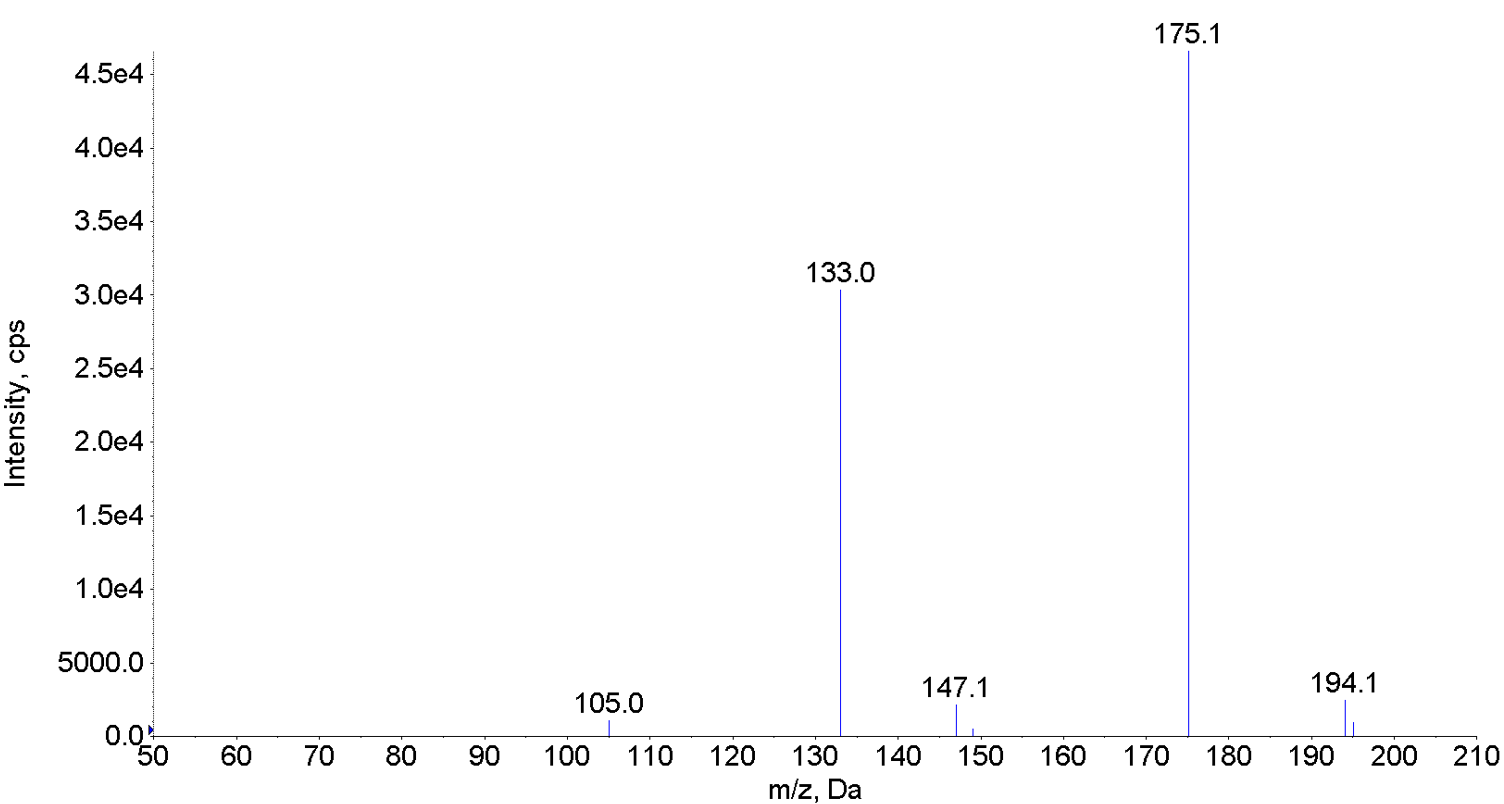


**Supplementary Figure 9.** MS^3^ spectrum of **M5**, product of *m/z* 342.2/195.1, excitation energy (AF2) 0.15.

## Radio-HPLC chromatograms of plasma samples after administration of (*S*)-[^18^F]1 in Humans


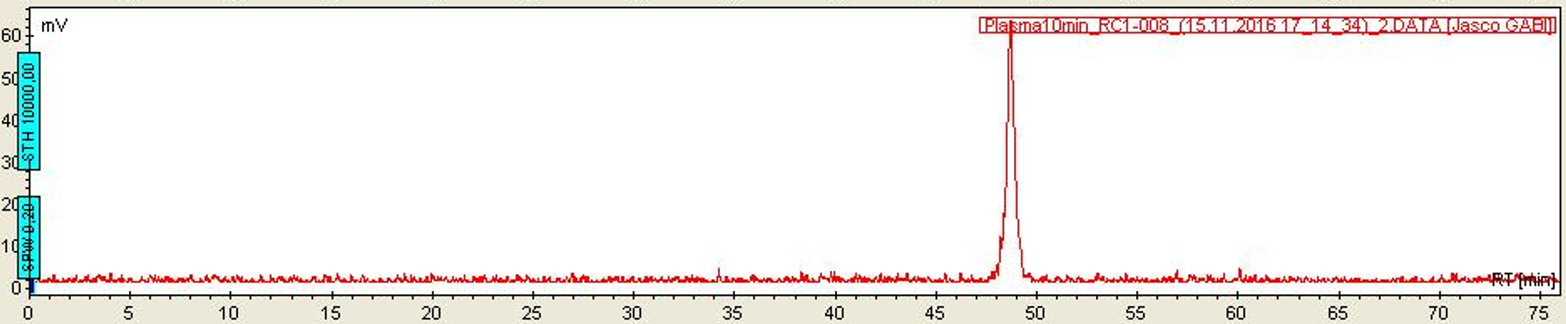


**Supplementary Figure 10.** Representative radio-HPLC chromatogram, plasma 10 min post injection (x axis: Time (min), y axis: Intensity γ detection (arbitrary units))


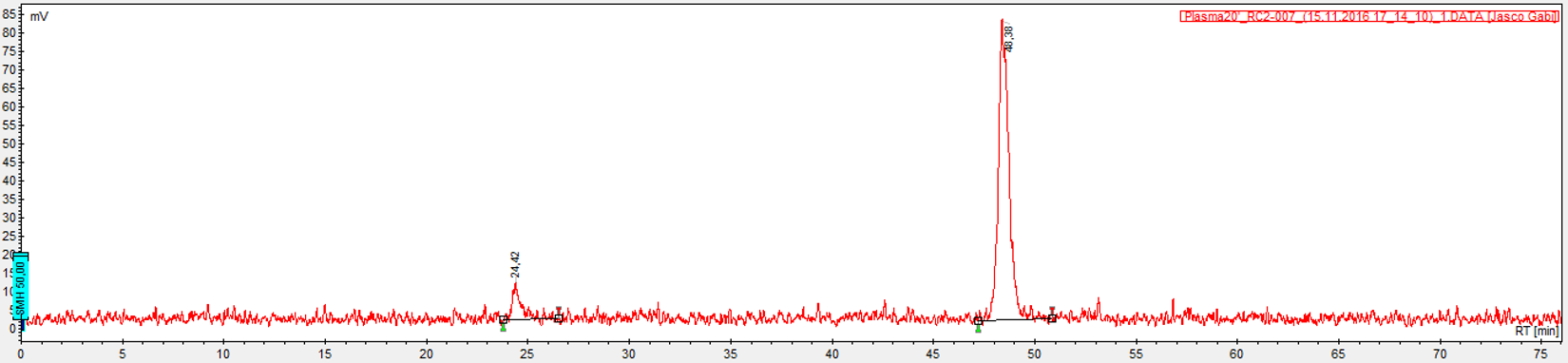


**Supplementary Figure 11.** Representative radio-HPLC chromatogram, plasma 20 min post injection (x axis: Time (min), y axis: Intensity γ detection (arbitrary units))


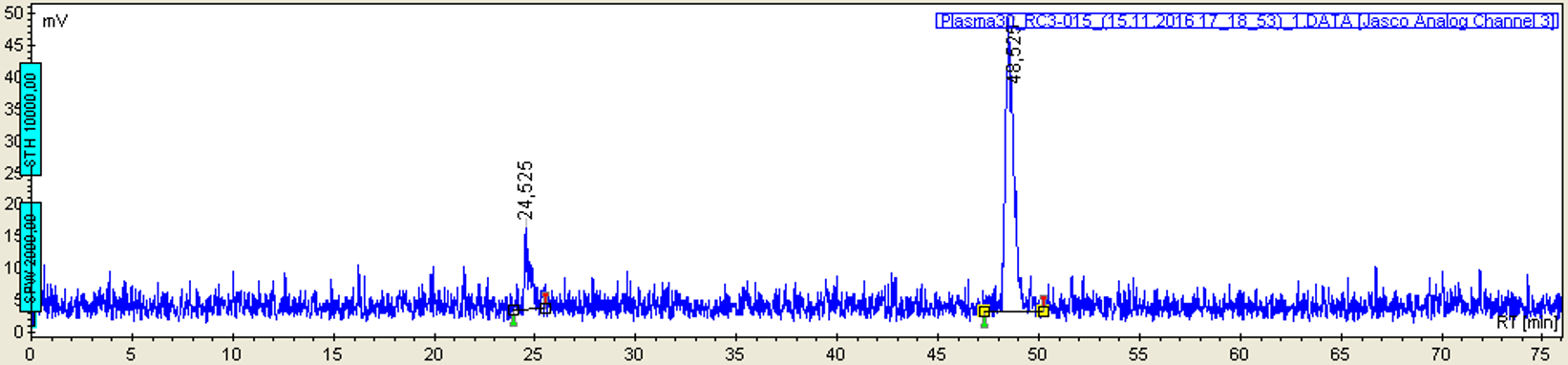


**Supplementary Figure 12.** Representative radio-HPLC chromatogram, plasma 30 min post injection (x axis: Time (min), y axis: Intensity γ detection (arbitrary units))
